# Early existence and biochemical evolution characterise acutely synaptotoxic PrP^Sc^

**DOI:** 10.1371/journal.ppat.1007712

**Published:** 2019-04-10

**Authors:** Simote Totauhelotu Foliaki, Victoria Lewis, Abu Mohammed Taufiqual Islam, Laura Jane Ellett, Matteo Senesi, David Isaac Finkelstein, Blaine Roberts, Victoria A. Lawson, Paul Anthony Adlard, Steven John Collins

**Affiliations:** 1 Department of Medicine (Royal Melbourne Hospital), The University of Melbourne, Parkville, Victoria, Australia; 2 Florey Institute of Neuroscience and Mental Health, Parkville, Victoria, Australia; 3 Department of Pathology The University of Melbourne, Parkville, Victoria, Australia; 4 Department of Microbiology and Immunology, The University of Melbourne, Parkville, Victoria, Australia; University of Alberta, CANADA

## Abstract

Although considerable evidence supports that misfolded prion protein (PrP^Sc^) is the principal component of “prions”, underpinning both transmissibility and neurotoxicity, clear consensus around a number of fundamental aspects of pathogenesis has not been achieved, including the time of appearance of neurotoxic species during disease evolution. Utilizing a recently reported electrophysiology paradigm, we assessed the acute synaptotoxicity of *ex vivo* PrP^Sc^ prepared as crude homogenates from brains of M1000 infected wild-type mice (cM1000) harvested at time-points representing 30%, 50%, 70% and 100% of the terminal stage of disease (TSD). Acute synaptotoxicity was assessed by measuring the capacity of cM1000 to impair hippocampal CA1 region long-term potentiation (LTP) and post-tetanic potentiation (PTP) in explant slices. Of particular note, cM1000 from 30% of the TSD was able to cause significant impairment of LTP and PTP, with the induced failure of LTP increasing over subsequent time-points while the capacity of cM1000 to induce PTP failure appeared maximal even at this early stage of disease progression. Evidence that the synaptotoxicity directly related to PrP species was demonstrated by the significant rescue of LTP dysfunction at each time-point through immuno-depletion of >50% of total PrP species from cM1000 preparations. Moreover, similar to our previous observations at the terminal stage of M1000 prion disease, size fractionation chromatography revealed that capacity for acute synpatotoxicity correlated with predominance of oligomeric PrP species in infected brains across all time points, with the profile appearing maximised by 50% of the TSD. Using enhanced sensitivity western blotting, modestly proteinase K (PK)-resistant PrP^Sc^ was detectable at very low levels in cM1000 at 30% of the TSD, becoming robustly detectable by 70% of the TSD at which time substantial levels of highly PK-resistant PrP^Sc^ was also evident. Further illustrating the biochemical evolution of acutely synaptotoxic species the synaptotoxicity of cM1000 from 30%, 50% and 70% of the TSD, but not at 100% TSD, was abolished by digestion of immuno-captured PrP species with mild PK treatment (5μg/ml for an hour at 37°C), demonstrating that the predominant synaptotoxic PrP^Sc^ species up to and including 70% of the TSD were proteinase-sensitive. Overall, these findings in combination with our previous assessments of transmitting prions support that synaptotoxic and infectious M1000 PrP^Sc^ species co-exist from at least 30% of the TSD, simultaneously increasing thereafter, albeit with eventual plateauing of transmitting conformers.

## Author summary

Although evidence clearly supports that misfolded prion protein (PrP^Sc^) is the principal component of “prions”, underpinning both transmissibility and neurotoxicity, consensus is lacking around the time of appearance and biochemical profile of neurotoxic species during disease evolution. Employing an electrophysiology model, measuring the capacity of brain homogenates derived from across the disease time-course to impair CA1 region long-term potentiation (LTP) and post-tetanic potentiation (PTP) in hippocampal slices, we observed that synaptotoxic species were present from 30% of the terminal stage of disease (TSD). Evidence that synaptotoxicity directly related to PrP species was demonstrated by significant rescue of LTP dysfunction at each time-point through immuno-depleting >~50% of total PrP species from cM1000 preparations. Moreover, size fractionation chromatography revealed that acute synpatotoxicity correlated with predominance of oligomeric PrP species in infected brains across all time points, while additional characterisation of cM1000 demonstrated that the predominant synaptotoxic PrP^Sc^ species up to and including 70% of the TSD were quite proteinase-sensitive. These findings in combination with our previous assessments of transmitting prions support that synaptotoxic and infectious M1000 PrP^Sc^ species co-exist from at least 30% of the TSD, simultaneously increasing thereafter, with biochemical transformation of synaptotoxic conformers continuing until late in disease.

## Introduction

Prion diseases are transmissible neurodegenerative disorders with human phenotypes including Creutzfeldt-Jakob disease (CJD), Gerstmann-Sträussler-Scheinker syndrome (GSS) and Kuru, while the principal animal diseases comprise scrapie in sheep and goats, bovine spongiform encephalopathy (“mad cow” disease) and chronic wasting disease in deer, elk and moose [1, 2]. The key pathogenic event in all prion diseases is believed to be misfolding of the normal prion protein (PrP^C^) into altered conformers (PrP^Sc^) with progressive accumulation of PrP^Sc^ in the brain linked to neurotoxicity through incompletely resolved mechanisms [3–8]. PrP^Sc^, especially that found at the terminal stage of disease (TSD: the advanced stage of disease requiring animal euthanasia) was previously construed as invariably highly protease-resistant whereas more recent evidence supports a broader spectrum encompassing a substantial proportion that are protease-sensitive [9–12], with such species evident during disease evolution [13] and terminal disease [10] most likely contributing to neurotoxicity [12].

Although experimental approaches exploiting successful rodent-adaptation of human and animal prion diseases such as CJD and scrapie have facilitated our understanding of the pathogenic evolution of these disorders [14–16], consensus around some fundamental aspects of pathogenesis has not been achieved. In contrast to various *in vivo* models consistently demonstrating rising titres of infectivity from early in disease development, well before overt clinical features [8, 12, 17] there is controversy over the time of appearance of neurotoxic PrP^Sc^ species. Reports of electrophysiological [18], morphological [19] and behavioural [20] disturbances prior to the mid-incubation point, generally coinciding with the first detection of PrP^Sc^, support relatively early production of neurotoxic PrP^Sc^. Conversely, other *in vivo* and *in vitro* studies describe that predominantly transmitting PrP^Sc^ species are produced first [21, 22] with neurotoxic species only propagated later in disease evolution nearer the onset of clinical disease, appearing to correlate with the plateauing of infectivity and depletion of PrP^C^ levels [12, 13, 21, 23]. Evidence of significantly increased expression levels of GFAP and active caspase 3, as well as heightened oxidative stress from around the mid-incubation period [8, 24] further support the likely presence of neurotoxic PrP^Sc^ species extant from relatively early in disease evolution, considerably pre-empting the onset of conventional clinical signs such as weight loss, ataxia, and hind limb paresis [8, 25].

In addition to uncertainty regarding the time of occurrence and propagation sequence of neurotoxic PrP^Sc^ species across the disease incubation period, it remains unresolved whether such species are devoid of transmission capacity or harbour both pathogenic properties. Some findings support that disease transmissibility and neurotoxicity are at least partially disconnected and perhaps relate to separate PrP^Sc^ species [12, 21, 23, 26]. Part of this apparent disconnection however, may relate to the types of techniques deployed to detect neurotoxicity [27], as well as the innate neuroprotective mechanisms of the host, such as microglial activation [28, 29] and brain clearance capacity [30], which may successfully mollify neurotoxicity to sub-threshold levels thereby delaying features evincing the presence of neurotoxic PrP^Sc^ over a prolonged period. Compounding such difficulties is the current lack of detailed understanding of the biochemical and biophysical characteristics of PrP^Sc^ species directly underpinning transmissibility and neurotoxicity and whether such properties change over the course of disease evolution. Recently we reported that acutely synaptotoxic PrP^Sc^ species derived from brains at the TSD appear to be at least modestly proteinase K (PK)-resistant and oligomeric [31] while other groups utilising size fractionation and sedimentation velocity fractionation with terminal disease tissue suggest that the most efficient PrP^Sc^ species for disease transmission are small oligomers [32, 33]. The reported predominance of relatively protease-sensitive PrP^Sc^ species until late in the incubation period [12, 34], which appear to be oligomeric [34], supports that both transmitting and synaptotoxic species extant earlier in disease progression would be likely to display such characteristics. This observation broadly concurs with the limited available information suggesting that the most infectious species and conversion to overt symptomatic disease corresponds with an increasing relative presence of PrP^Sc^ conformers found in smaller, more protease-sensitive oligomers [13]. Also to be determined is whether there is a single or predominant neurotoxic PrP^Sc^ species and if so, does specific biochemical or biophysical transformation occur during disease progression or conversely does a diverse spectrum of toxic PrP^Sc^ of varied biochemical properties eventuate during disease progression.

As a part of studies comprehensively characterising a new electrophysiological paradigm, we recently demonstrated the ability to objectively and sensitively detect the presence of acutely synaptotoxic *ex vivo* PrP^Sc^ derived from brains of mice terminally ill with M1000 prion infection [31]. The current study aimed to determine whether such synaptotoxic species could be detected in the brains of mice during disease evolution following precise stereotaxic intracerebral inoculation with M1000 prions. To achieve this, we assessed the acute synaptotoxicity of *ex vivo* homogenate preparations derived from the brains of M1000 infected mice (cM1000) at 30%, 50%, 70% and 100% of the TSD, through exploring the capacity of cM1000 to impair hippocampal CA1 region long-term potentiation (LTP), paired-pulse facilitation (PPF), and post-tetanic potentiation (PTP) in explant hippocampal slices. LTP and PTP are physiological measures of synaptic plasticity correlating with memory and learning induced by repetitive high frequency stimulations wherein PTP is a short-term predominantly pre-synaptically mediated enhancement of synaptic responsiveness followed by LTP, which is expressed as a persistently enhanced post-synaptic potential generated by a combination of both pre- and post-synaptic functions [31]. PPF is a physiological measure of the probability of neurotransmitter release (*Pr*), which is frequently used to estimate *Pr* during expression of LTP [31]. Of particular note, cM1000 from 30% of the TSD was able to cause significant impairment of LTP, PPF, and PTP. Evidence that the synaptotoxicity directly related to PrP species was demonstrated by the significant rescue of LTP dysfunction at each time-point through immuno-depletion of >50% of total PrP species from cM1000 preparations. Size fractionation chromatography revealed that capacity for acute synaptotoxicity correlated with predominance of oligomeric PrP species in infected brains across all time points, with the profile appearing maximised by 50% of the TSD. Interestingly, both pooled oligomeric and monomeric PrP^Sc^ fractions from across the time-points were acutely synaptotoxic, with the toxicity of pooled monomeric fractions appearing associated with likely rapid spontaneous oligomerization of PrP^Sc^ monomers in physiological buffer following their size fractionation. Moreover, the synaptotoxicity of cM1000 from 30%, 50% and 70% of the TSD, but not at 100% TSD, was abolished by digestion of immuno-captured PrP species with mild PK treatment (5μg/ml for an hour at 37°C), demonstrating that the predominant synaptotoxic PrP^Sc^ species up to and including 70% of the TSD appears quite proteinase-sensitive. Overall, these findings in combination with our previous assessments of transmitting prions support that synaptotoxic and infectious M1000 PrP^Sc^ species co-exist from at least 30% of the TSD.

## Materials and methods

### Ethics statement

All animal handling was in accordance with National Health and Medical Research Council (NHMRC) guidelines. Animal handling and experimental procedures were approved by The Florey Institute of Neuroscience and Mental Health Animal Ethics Committee (Ethics number: 13–048) or the Biochemistry & Molecular Biology, Dental Science, Medicine (RMH), Microbiology & Immunology, and Surgery (RMH) Animal Ethics Committee, The University of Melbourne (Ethics number: 1312997.1).

### Animals

The M1000 prion strain has been well described and characterised [8], and was originally adapted to mice from a person most likely dying from GSS [15]. Mice used for electrophysiological studies and to generate M1000 infected brains at varying stages of prion infection following stereotaxic intracerebral (ic) inoculation were 12-week-old wild-type (WT) female C57BL/6J mice (Animal Resource Centre, Western Australia). In addition, 12-week-old WT female Balb/c mice were used to generate M1000 infected brains through routine ic inoculation as described previously [8]. Mice used for bioassay studies were six-week old transgenic (over-expressing PrP^C^ ~10-fold; [35]) tga20 mice bred at the Florey Institute of Neuroscience and Mental Health (originally a generous gift from The Scripps Research Institute, La Jolla, California, USA).

### Mice inoculations for electrophysiology experiments

To optimise precision with M1000 inoculations for producing time course *ex vivo* preparations for electrophysiology experiments, 12-week old WT C57BL/6J mice were stereotactically ic inoculated. Briefly, mice were anesthetized with an intraperitoneal cocktail injection of ketamine (100mg/kg) and xylazine (20mg/kg) and placed on a small animal stereotactic frame (Model 940, Kopf, Germany fitted with a Model 5000 microinjection unit) equipped with a 37°C heating mat. The pedal reflex was monitored every 15–20 minutes to assess the level of unconsciousness. A small incision along the sagittal line of the scalp allowed the identification of the bregma. A small drill fitted with a blunt burr was then used to make a hole in the skull, through which a 26-gauge needle (Model 1701, Hamilton, Switzerland) was placed. Four microlitres of 10% (w/v in sterile PBS) cM1000 was then injected (flow-rate of 0.8μl/min) just above the dorsal hippocampus (bregma coordinates: -2.5 caudally, +/- 2.5 laterally and -1.5 ventrally). The needle was then slowly retracted, placed in position above the contralateral hippocampus and the procedure repeated as above. The scalp wound was closed with Super Glue and Ilium Neocort was applied. The mice were allowed to recover for 1 hour on a 37°C heating mat and then transferred to single-caging for 3 days. The mice were checked once daily for physiological parameters such as weight and normal motor behaviour and an intraperitoneal dose of carprofen (5mg/kg) given at 0, 24 and 48 hours after surgery to relieve pain. These M1000 inoculated mice reach the TSD (requiring euthanasia) at a mean of ~170 (±3 SD) days post-inoculation (dpi). Relative to the TSD, mice were sacrificed at 30%, 50%, 70% and at the 100% time points (ie representing ~51, ~85, ~119, and ~170 dpi, respectively), brains were collected and homogenized to 20% (w/v) in 1x PBS, and stored at -80°C until use. As negative or sham controls, similar aged WT mice were ic inoculated as above with normal brain homogenate (NBH; 10% w/v in PBS) and brains were harvested at the same dpi as the M1000 infected mice. To further assess the presence of synaptotoxic PrP^Sc^ species at an early time point following M1000 inoculation, 12-week-old WT female Balb/c mice were routinely ic inoculated as described previously [36] with brains collected at 30% of the TSD (representing ~44 dpi).

### Immuno-depletion/-precipitation of PrP

Total prion proteins were specifically immuno-depleted using 03R19 anti-PrP polyclonal antibody from 1% (w/v) cNBH and cM1000 as previously described [31] to generate depleted normal and M1000 brain homogenates (dNBH and dM1000, respectively), with specificity checked by comparison with “mock” immuno-depletion using normal rabbit serum. Additionally, the immuno-precipitated prion proteins were eluted from protein-G-sepharose pellets reconstituted to original volumes, by a mild PK treatment of 5 μg/mL at 37°C for an hour, with two cycles of 20-minutes of agitation interspersed with 10 minutes without agitation to generate PK-eluted immuno-precipitated NBH and M1000 brain homogenates (PK+IP-NBH and PK+IP-M1000, respectively). Aliquots of the ~1% (w/v) depleted and resuspended PK-treated preparations were taken for analysis by western blotting, prior to further dilution to 0.5% (w/v) in artificial CSF (aCSF: 126mM NaCl, 2.5mM KCl, 26mM NaHCO_3,_ 1.25mM NaH_2_PO_4,_ 10mM Glucose, 1.3mM MgCl_2_.6H_2_O and 2.4mM CaCl_2_.2H_2_O) for use in electrophysiology studies.

### Electrophysiology paradigm

Electrophysiology studies were performed as described [31] with brain homogenates for these studies pre-cleared by a one minute 100xg spin. In brief, 300μm hippocampal slices were prepared from WT mice utilizing a vibratome (Leica VT1200S) and ice cold cutting solution (3mM KCl, 25mM NaHCO_3,_ 1.25mM NaH_2_PO_4,_ 206mM Sucrose, 10.6mM Glucose, 6 mM MgCl_2_.6H_2_O, 0.5mM CaCl_2_.2H_2_O) while continuously carboxygenating with 5% CO_2_ and 95% O_2_. Following an hour of incubation in carboxygenated aCSF at 32°C, slices were mounted on to 60MEA200/30iR-Ti-pr-T multi-electrode arrays (MEA; Multichannel Systems; Germany) for recording while continuously superfused with carboxygenated aCSF. Harp slice grids (ALA HSG-5B, Multichannel Systems; Germany) were utilized to ensure optimal contact of the slices with microelectrodes. Electric stimulation (1500 to 2500 mV) was utilised to evoke hippocampal field excitatory post-synaptic potentials (fEPSP) from the Schäffer collateral pathway while recording from the CA1 region. A 30-minute baseline was recorded at 30 second intervals using a basal stimulus determined by an input-output curve, which was obtained by stimulating the Schäffer collateral pathway with increasing stimulation intensities (at 30 second intervals) starting from 500 mV to a stimulation intensity that evoked the maximum fEPSP as indicated by plateau curve (usually between 4000–5000 mV) wherein the basal stimulus was declared as the stimulus that evoked ~40% of the maximum fEPSP. Channels of the MEA grid utilized for analysis were those best aligned to the Schäffer collateral pathway through recording from electrodes placed on the stratum radiatum of the CA1 region and selecting those demonstrating fEPSPs that manifested PPF (as described previously [31]), generated positive trend input-output curves, produced non-spiky fEPSP curves, and maintained fEPSP amplitudes above the eight standard deviation threshold of the noise levels for at least 80% of the total recording time. The average number of electrodes recorded from and utilised for analysis in each slice was seven. The treatments (the crude homogenates or other preparations) were superfused over the hippocampal slices for five minutes following 8–10 minutes of stable baseline. For tetanus, three trains of high frequency stimulation (HFS: 100Hz each) were applied (for 0.5 seconds at 20 second intervals) following the baseline recording to induce PTP and LTP. Each train of HFS evoked serial pulses of fEPSPs, wherein the first nine of those pulses were recorded to estimate presynaptic activity associated with the induction of PTP. Post-HFS recording continued for 30 minutes wherein the first response was utilised to estimate PTP and last 10-minute period taken as representing LTP. LTP and PTP were calculated as the percentage fEPSP increase after HFS relative to the last five-minute baseline of fEPSPs. PPF was evoked by basal stimuli delivered 20 ms apart as previously reported [31]. The ratio between the fEPSP amplitude of the first and the second pulse was the PPF ratio. PPF ratio was measured before treatment and induction of LTP (PPF1) and after treatments and expression of LTP (PPF2).

### Incubation time interval bioassays

To assess levels of prion infectivity contained within the various *ex vivo* M1000 PrP^Sc^ preparations utilized to characterise acutely synaptotoxic species present in cM1000 derived from terminal prion disease, tga20 mice were ic inoculated with these *ex vivo* samples (30μL per mouse) as previously described [8]. In addition, 1% (w/v) cM1000 and NBH served as positive and negative controls, respectively. The tga20 mice were euthanized when they become terminally ill as indicated by features including reduced spontaneous activity, prominent ataxia and hind limb paresis. Total survival in dpi was recorded and utilized to calculate an approximate, especially relative infectivity titre for each *ex vivo* M1000 PrP^Sc^ preparation as described [8]. One-way ANOVA was used to compare the average terminal incubation period of tga20 mice, as well as average infectivity titre of inocula. The infectivity titres, ID_50_ units/g brain were converted to log ID_50_ units/μL 1% crude M1000 brain homogenate and plotted as a function of percentage of incubation period to the terminal stage.

### Western blotting

PrP levels were analysed by standard PAGE and immunoblotting as described previously [37], with protease-resistant PrP also detected following the higher sensitivity method of sodium phosphotungstate (NaPTA) precipitation as previously described [36]. For non-NaPTA immunoblotting, brain homogenates (1% w/v) from all time points were either not treated or treated with 5μg/mL PK for an hour at 37°C, while brain homogenates undergoing NaPTA precipitation were treated with 5, 25, and 50μg/mL PK for an hour at 37°C. Proteins were denatured in 1x sample buffer (containing 6% Beta-mercaptoethanol), resolved on NuPAGE Novex 4–12% Bis-Tris gels (ThermoFisher Scientific), transferred to PVDF membrane (Millipore; transfer buffer containing 25 mM Tris, 200 mM glycine and 20% methanol), blocked with 5% (w/v) skim milk then probed with the anti-PrP primary antibodies either 03R19[14] or 8H4 (Abcam) as indicated in the figure legends. For native western blotting, protein samples containing 2% (w/v) Sarkosyl were diluted 1:1 with 2x Novex Tris-Glycine native sample buffer, resolved on NuPAGE Novex 3% Tris-Acetate gels in 1x Novex Tris-Glycine native running buffer (at constant 160V; ThermoFisher Scientific), and transferred to PVDF membrane (at constant 70V for 3 hours) using a transfer buffer containing 25 mM Tris, 200 mM glycine and 10% methanol. A few microliters of a High Marker protein standard (Invitrogen) were resolved alongside the protein samples on native gels to estimate sizes of native proteins. The PVDF membranes of native protein samples were blocked and probed with 8H4 antibody to detect PrP species. Following the appropriate secondary antibody, protein bands were detected by chemiluminescence (ECL Prime and Select, Invitrogen) and digitized in a Fujifilm LAS-3000 Intelligent dark box. Membranes were stained with Coomassie blue to determine relative total protein levels. Protein bands of interest were quantified by densitometry (Image J) normalized for total protein level as previously described [37]. Approximate estimation of PrP quantities in preparations used for electrophysiology studies was achieved by intra-experimental comparison to a recombinant full-length mouse PrP (rPrP) standard curve, as described previously [31].

### Size exclusion chromatography

As described in detail previously [31], whole brains from infected mice at each time point, as well as sham-infected control mice, were homogenized at 20% (w/v in 1x PBS), pelleted at a 15000xg spin, solubilized with 4% (w/v in 1x PBS) Sarkosyl into ~10% (w/v) final concentration, and centrifuged at 10000xg to collect the supernatants. The supernatants were then exhaustively dialyzed using 10 kDa cut-off dialysis membranes in 1x PBS dialysate (without calcium) and filtered across a 0.22-micron filter. Approximately 3 mL of each preparation was slowly injected into a sephacryl-100 column pre-equilibrated with at least 2 column volumes of 1x PBS containing calcium and magnesium. The protein complexes were eluted in 1x PBS (containing calcium and magnesium) at a flow rate of 0.5 mL per minute, wherein the void volume was collected at ~70 minutes after injection. One mL fractions were then collected every two minutes for 80 minutes (40 fractions) following collection of the void volume. The relative levels of PrP in each fraction were analysed by western blotting, including before and after digestion with PK as described [31]. The size of proteins or complexes eluted into each fraction was determined through size fractionation of size exclusion chromatography protein markers: bovine erythrocyte carbonic anhydrase (~29kDa), bovine serum albumin (~66kDa), yeast alcohol dehydrogenase (~150kDa), sweet potato beta-amylase (~200kDa), horse spleen apoferritin (~443kDa), and bovine thyroglobulin (~669kDa) [31]. Operationally, PrP species eluted into fractions with molecular weights smaller than 100kDa were considered to be mostly monomeric PrP while prion proteins eluted into fractions with molecular weights >100kDa but <500kDa were considered predominantly oligomeric PrP or large protein complexes containing PrP and although not experimentally verified PrP >500kDa was considered to represent increasingly fibrillar assemblies.

### Statistical analyses

The total levels of PrP and levels of at least modestly PK resistant PrP^Sc^ were compared across time points of the disease evolution by One-way ANOVA. Levels of PK-resistant PrP^Sc^ across the time points detected by NaPTA precipitation after digestion with each PK concentration (5, 25, and 50μg/mL) were compared by One-way ANOVA with Tukey’s correction for multiple comparisons. The average LTP or PTP in slices treated with M1000 preparations from across the time points were compared with the appropriate negative controls by One-way ANOVA with Dunnett’s correction for multiple comparisons (comparing mean LTP and PTP of M1000 treated slices to those of negative controls). The degree of synaptotoxicity was calculated as the percentage LTP reduction from that observed in negative controls. The average degree of synaptotoxicity as either LTP or PTP disruption in slices treated with M1000 preparations from across the time points were compared by One-way ANOVA with Tukey’s correction for multiple comparisons (comparing mean degree of toxicity at each time point with that at every other time point). Unpaired Student t-test was used to compare the average PrP levels before and after the PrP-immuno-depletion. PPF was calculated as the percentage PPF ratio decrease in PPF2 relative to PPF1 and compared to that of the appropriate negative control by One-way ANOVA with Dunnett’s correction for multiple comparisons. The probability of neurotransmitter release during HFS trains was calculated by dividing the second pulse by the first pulse of the first 9 pulses evoked by each train. The average probability of neurotransmitter release at the second and third train was compared to that of the first train by One-way ANOVA with Dunnett’s correction for multiple comparisons. The depletion of the readily releasable pool (RRP) of neurotransmitter during each train of HFS was estimated and compared between treatment groups using a one-phase decay exponential function to determine the slope of fEPSP amplitude decline (time constant of decay) from pulse three to the last pulse as described previously [31]. The size of RRP was estimated and compared between treatment groups by a linear fit equation of cumulative fEPSP of each train (best fit of the last four cumulative fEPSP) wherein the size of RRP was the Y-intercept of the linear fit (see Methods of [31] for further details). The efficiency of RRP replenishment following each HFS train was determined by size of RRP increase at train two and three [31]. One-way ANOVA with Tukey’s correction for multiple comparisons was used to compare the average incubation period of tga20 mice inoculated with different preparations from the TSD, as well as to compare the average infectivity titres of these M1000 preparations. Tukey’s correction for multiple comparisons was used to compare the mean of each group to the mean of every other group (suited for time point results), whereas Dunnett’s correction for multiple comparison was used to compare the mean of the control group to the mean of every other tested group.

## Results

### Acutely synaptotoxic PrP^Sc^ is detectable from early in M1000 prion disease development

**Fig 1:**
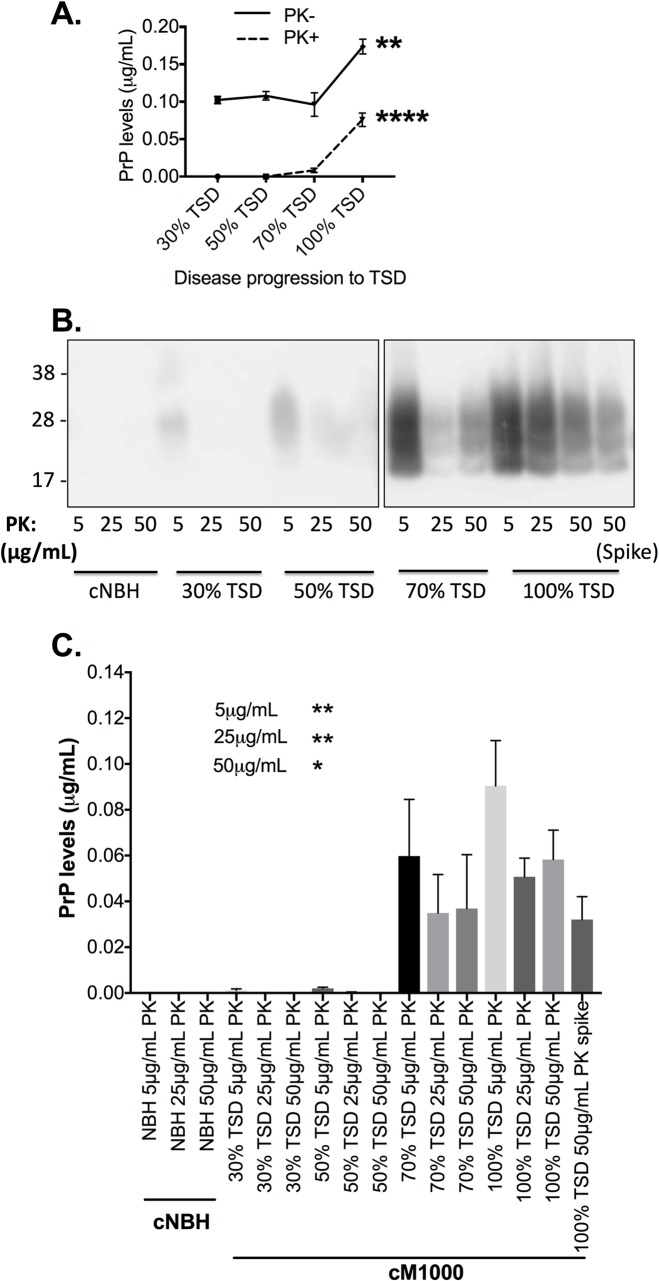
Levels of total PrP and proteinase K (PK)-resistant PrP^Sc^ species across the natural evolution of M1000 prion disease. (A) Quantification of the immunoblots in S1A Fig with the levels of total PrP (without PK digestion: PK-) and at least modestly PK-resistant PrP^Sc^ (PK+) compared across M1000 disease evolution by One-way ANOVA with Tukey’s correction for multiple comparisons. (B) Western immunoblots of cNBH and cM1000 from all the disease time points following precipitation of PrP^Sc^ species by sodium phosphotungstic acid (NaPTA) and digestion with 5, 25, and 50 μg/mL PK (at 37°C for an hour), respectively. The “spike” represents the positive control demonstrating successful precipitation and detection of PrP^Sc^ species in 5μL of 10% (w/v) cM1000 that was spiked into 500μL of 1% (w/v) cNBH. (C) Approximate quantification of PrP levels (in μg/mL) in brain homogenates in B (n = 4). Results are presented as mean ± standard error of mean. *p<0.*05*, **p<0.*01*, ****p<0.*0001*.

Neuropathological features of prion disease such as microvacuolation and astrocytic gliosis are initially detected in the hippocampus and thalamus of WT Balb/c mice ic infected with M1000 prions at ~57% of the TSD (~83 dpi) [8], which suggests the presence of neurotoxic PrP^Sc^ species prior to this. To explore whether acutely synaptotoxic PrP^Sc^ species are propagated at earlier time points of M1000 prion disease pre-empting these first neuropathological changes, we used a recently developed electrophysiology paradigm to assess cM1000 prepared from WT mice at 30%, 50%, 70% and 100% of the TSD following stereotaxic ic infection with M1000 prions. To biochemically characterise the 0.5% (w/v) cM1000 from each of the time points (n = 3 for each time point) they were initially evaluated by routine western blotting before and after treatment with modest PK digestion in combination with comparative quantitative analysis based on a rPrP standard curve. A significant increase in total PrP (*p* = 0.0019), largely due to increased levels of at least modestly PK-resistant PrP^Sc^ (*p*<0.0001) at 100% of the TSD relative to earlier time points was observed (Fig 1A & S1A Fig) with a trend towards reduced total levels at 70% of the TSD. Moreover, at least modestly PK-resistant PrP^Sc^ was not detected at 30% and 50% of the TSD, with only minimal levels evident at 70% of the TSD (~0.005 μg/mL), which were approximately 16-fold less than the robust levels observed at 100% of the TSD (~0.080 μg/mL). As expected, PK-resistant PrP was never observed in cNBH across the time course, with no difference in PrP^C^ levels across all time points (S1B Fig).

To further characterise the PrP in the 0.5% (w/v) NBH and cM1000 across the time course, western blotting following NaPTA precipitation and digestion with increasing PK concentrations was performed. In cM1000 preparations, this analysis (Fig 1B & and 1C) revealed barely detectable levels of very modestly PK-resistant PrP^Sc^ at 30% (<< 0.012μg/mL) and 50% (<<0.012 μg/mL) of the TSD, with significantly higher levels at 70% (~0.059 μg/mL) and 100% (~0.090 μg/mL) of the TSD (*p* = 0.0043; One-way ANOVA with Tukey’s correction for multiple comparisons). The 25 μg/mL PK digestion completely abolished PK-resistant PrP^Sc^ species at 30% of the TSD, while minimal levels were detected at 50% (<<0.012μg/mL) of the TSD and significantly higher levels observed at 70% (~0.035 μg/mL) and 100% (~0.051 μg/mL) of the TSD (*p =* 0.0045; One-way ANOVA with Tukey’s correction for multiple comparisons). The 50 μg/mL PK completely digested PK-resistant PrP^Sc^ species at 30% and 50% of the TSD, while significantly higher levels remained at 70% (~0.037 μg/mL) and 100% (~0.058 μg/mL) of the TSD (*p =* 0.023; One-way ANOVA with Tukey’s correction for multiple comparisons). Of note, these data suggest that PK-resistant PrP^Sc^ species at 70% of the TSD were rendered more detectable by NaPTA precipitation compared to results of routine western blotting such that levels of PK-resistant PrP^Sc^ species (including at least modestly PK-resistant species) were no longer significantly different to those at 100% of the TSD (compare Fig 1B and 1C with S1A Fig). Given PK-resistant PrP was never observed in cNBH across the time points, only cNBH from 100% of the TSD was utilized as negative control for assessing acute synaptotoxicity.

**Fig 2:**
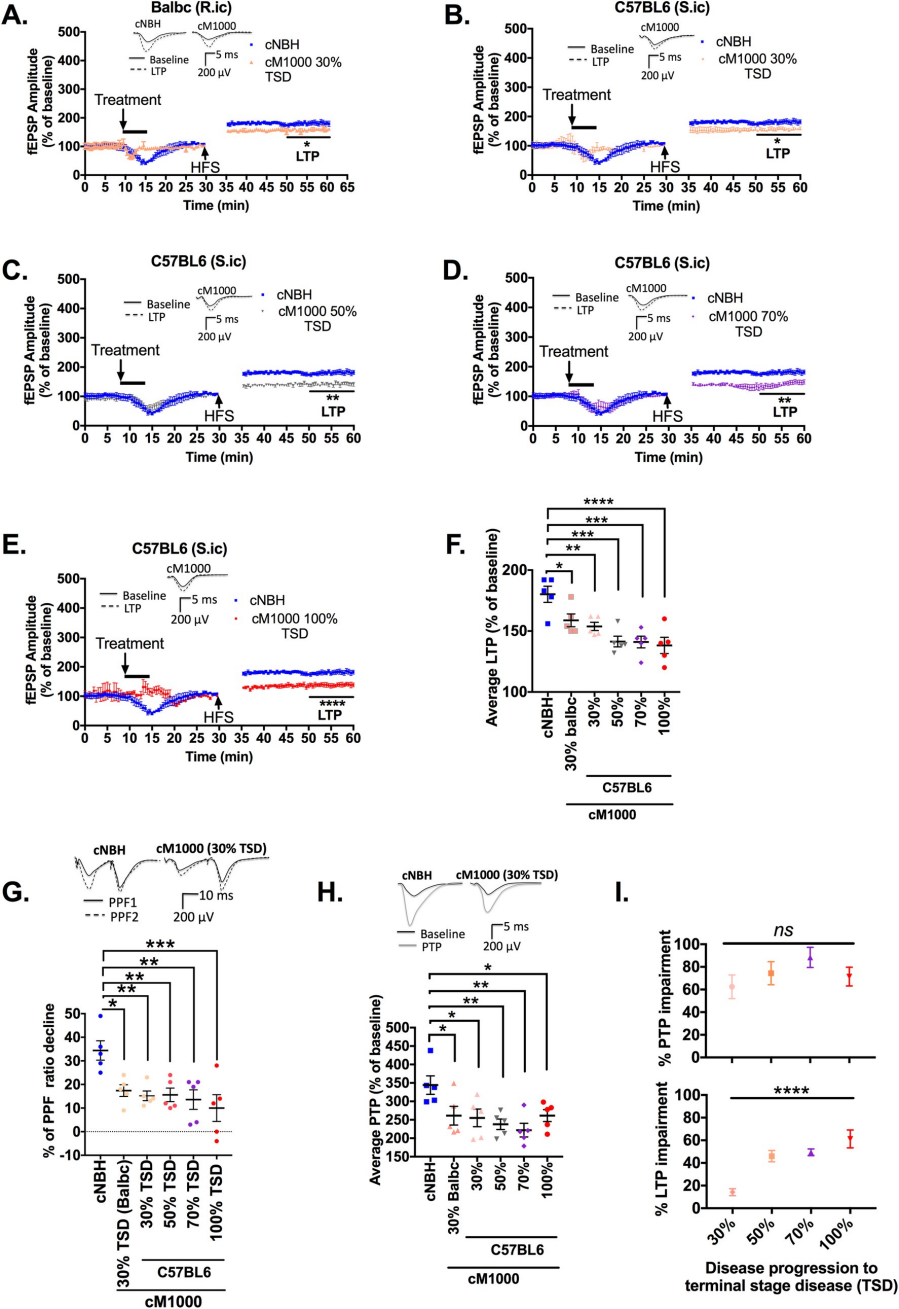
Synaptotoxic PrP^Sc^ species are propagated from early in the evolution of M1000 prion disease. (A) CA1 region LTP of WT mouse hippocampal slices was significantly disrupted following a five minute exposure to 0.5% (w/v; in artificial CSF) crude M1000 brain homogenates (cM1000) derived from M1000 infected WT Balb/c mice (infected through routine intracerebral injection [R.ic]) at 30% of the incubation period to the terminal stage of disease (TSD). (B to E) CA1 region LTP of WT mouse hippocampal slices was significantly disrupted following a 5 minute exposure to 0.5% (w/v; in artificial CSF) cM1000 derived from M1000 infected WT C57BL/6J mice (infected by stereotaxic intracerebral injection [S.ic]) at 30% (B), 50% (C), 70% (D) and 100% (E) of the TSD. (F) Average LTP from the last 10 minutes of recordings (fEPSP readings following high frequency stimulation, HFS) after treatments with cM1000 from 30%, 50%, 70% and 100% of the TSD compared to the negative control 0.5% (w/v) crude normal brain homogenates (cNBH) derived from sham infected mice at the equivalent of 100% of the TSD. (G) Average percentage paired pulse facilitation (PPF) ratio decline following treatments in WT mouse hippocampal slices treated with cM1000 compared with cNBH controls as described in the Methods. (H) The average PTP generated by WT mouse hippocampal slices treated with cM1000 from across the disease evolution compared to the cNBH control as described in the Methods. (I) The percentage of PTP and LTP impairments (upper and lower panel, respectively) following treatments with cM1000 from each time point of the disease progression calculated as the percentage of PTP and LTP decrease compared to the normal PTP and LTP in slices treated with cNBH controls (One-way ANOVA with Tukey’s correction for multiple comparison). (A to E) The five minute treatments started after 8-to-10 minutes of stable baseline. The HFS trains were applied following 30 minutes of baseline recordings. (A, C, D) Results are presented as mean ± standard error of mean. (A-E, F, G & H) Average LTP/PTP/PPF ratio degree of decline in WT mouse hippocampal slices treated with cM1000 preparations from across the time points were compared with NBH controls by One-way ANOVA with Dunnett’s correction for multiple comparisons. (A-E, G & H) Some examples of raw fEPSP traces are provided as insets. *p<0.*05*, **p<0.*01*, ***p<0.*001*, ****p<0.*0001*, *ns* = not statistically significant (*p>0*.*05*).

Relative to the normal CA1 region LTP obtained in WT hippocampal slices treated with cNBH (180 ± 7%; n = 5), the CA1 LTP was significantly impaired following exposure to cM1000 prepared from the brains of C57BL/6J mice at 30% of the TSD following M1000 prion inoculation through hippocampal stereotaxic injection (154 ± 4%; *p = 0*.*0122*; n = 5; Fig 2B; One-way ANOVA with Dunnett’s correction for multiple comparisons), which was very similar to the degree of LTP disruption obtained in WT hippocampal slices after exposure to cM1000 derived from brains of routinely ic inoculated Balb/c mice also culled at 30% of the disease progression (159 ± 5%; *p = 0*.*0368*; n = 5; Fig 2A; One-way ANOVA with Dunnett’s correction for multiple comparisons). CA1 LTP was also significantly impaired by cM1000 derived from the brains of stereotactically infected C57BL/6J WT mice at 50% (142 ± 4%; *p = 0*.*0001*; n = 5), 70% (141 ± 5%; *p = 0*.*0001*; n = 5) and 100% (133 ± 6%; *p*<*0*.*0001*; n = 5) of the TSD (Fig 2C–2F; One-way ANOVA with Dunnett’s correction for multiple comparisons). Consistently, the degree of PPF ratio decline in slices with impaired LTP were significantly lower than those in slices treated with cNBH demonstrating normal LTP, confirming significant disruption of the probability of neurotransmitter release associated with LTP dysfunction (Fig 2G; One-way ANOVA with Dunnett’s correction for multiple comparisons). In addition, PTP was significantly disrupted in WT slices treated with cM1000 prepared from each time point (Balb/c at 30% of the TSD, 261 ± 25%, *p = 0*.*0480*; C57BL/6J at 30% of the TSD, 255 ± 24%; *p = 0*.*0334*; 50% of the TSD: 238 ± 14%, *p = 0*.*0093*; 70% of the TSD, 222 ± 19%, *p = 0*.*0052*; and 100% of the TSD: 261 ± 25%, *p = 0*.*0191*; n = 5 for each time point) relative to normal PTP generated in WT hippocampal slices treated with cNBH (344 ± 25%; n = 5; Fig 2H). Interestingly, the degree of PTP impairment did not significantly increase across the time points (*p = 0*.*3306*; One-way ANOVA with Tukey’s correction for multiple comparisons; Fig 2I upper panel), whereas the degree of LTP impairment significantly increased across the disease evolution in mice inoculated by stereotaxic injection (*p<0*.*0001*; One-way ANOVA with Tukey’s correction for multiple comparisons; Fig 2I bottom panel). Transient depression of fEPSPs during and following the brief treatments was frequently observed, but importantly, they invariably return to baseline before instigation of HFS trains, with potential explanations for this phenomenon and the lack of implications for assessments of synaptic function discussed in detail previously [31].

Further analyses of presynaptic events during HFS trains that subsequently induced PTP were undertaken. Of note, the cNBH negative controls used in these electrophysiology studies did not affect synaptic functions as compared to the aCSF technical controls (S2A–S2G, S2K & S2L Fig). Specifically, normal RRP replenishment was exhibited by slices treated with aCSF and cNBH wherein the size of RRP significantly increased at train 2 and again at train 3 (S2K & S2L Fig); however, the RRP replenishment was impaired in slices with PTP impairment, wherein the RRP size failed to significantly increase from train 2 to train 3 across all treatments with cM1000 from the time course (S2M–S2P Fig). Although the RRP replenishment was impaired, the probability of neurotransmitter release was normal with the second and third trains evoking significantly higher neurotransmitter release than the first train (S2D Fig). The RRP rate of depletion was also normal, whereby the time-constant of decay of the third-to-ninth pulses were not different when comparing between slices treated with cNBH and those treated with cM1000 (S2H–S2J Fig).

### Acute synaptotoxicity derived from earlier time points of M1000 prion disease evolution is associated with PrP species

**Fig 3:**
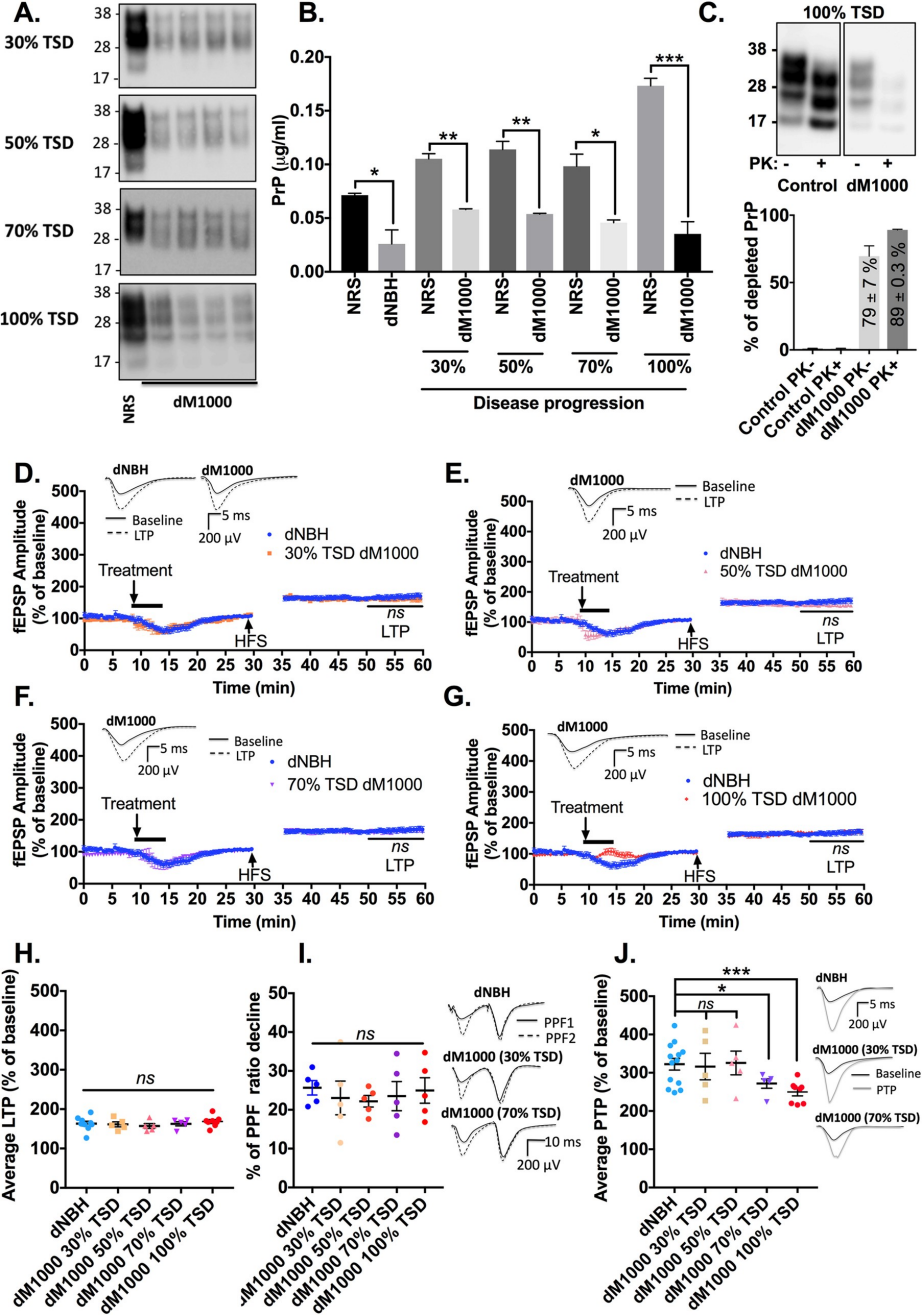
Acute synaptotoxicity of cM1000 from earlier time points of M1000 prion disease is associated with prion protein species. (A) Western blotting of 20 μL 1% (w/v) crude M1000 brain homogenates (cM1000) from 30%, 50%, 70%, and 100% of M1000 prion disease progression to terminal stage disease (TSD) following mock immuno-depletion of total PrP species with normal rabbit serum (control) or immuno-depletion of total PrP species with 03R19 prion antibody (Probed with 8H4 antibody; molecular weight markers are provided at left). (B) Quantification of total PrP species in PrP immuno-depleted normal brain homogenate (dNBH) and PrP immuno-depleted cM1000 (dM1000) from across the disease progression (by densitometry analysis) compared to appropriate negative controls by Unpaired Student’s t-test (mock immuno-depleted NBH or mock immuno-depleted cM1000). (C) Upper panel: A representative western blot of PrP levels in 20 μL cM1000 at 100% TSD following a mock immuno-depletion with normal rabbit serum (NRS) and an immuno-depletion with 03R19 antibody, and mild PK digestion. Lower panel: Quantification of total PrP (n = 4) and at least modestly PK-resistant PrP (n = 2) immuno-depleted from cM1000 (from 100% of the TSD) by 03R19 relative to the controls (probed with 8H4 antibody; molecular weight markers are provided at left). (D to G) LTP of WT mouse hippocampal slices following a five minute treatment with 0.5% (w/v in aCSF) dM1000 from 30% (D), 50% (E), 70% (F), and 100% of the TSD (G) compared with the LTP of slices treated with 0.5% (w/v in aCSF) dNBH controls (One-way ANOVA with Dunnett’s correction for multiple comparisons). (H) The average LTPs, (I) the average percentage of PPF ratio decline, and (J) the average PTPs generated by WT mouse hippocampal slices treated with dNBH and dM1000 from across time points of the disease progression were compared by One-way ANOVA with Dunnett’s correction for multiple comparisons when comparing across time points. (D to G) The five minute treatment started after 8-to-10 minutes of stable baseline. The high frequency stimulation (HFS) trains were applied following 30 minutes of baseline recordings. Results are presented as mean ± standard error of mean. (D-G, I & J) Some examples of raw fEPSP traces are provided as insets. *p<0.*05*, **p<0.*01*, ***p<0.*001*, *ns* = not statistically significant (*p>0*.*05*).

To determine if *ex vivo* PrP species propagated at earlier time points of the disease evolution are directly associated with the acute synaptotoxicity of cM1000, we immuno-depleted total PrP species in cM1000 derived from each time point and assessed the acute synaptotoxicity of the depleted M1000 preparations using our electrophysiology paradigm. The immuno-depletion removed ~63 ± 18% of total PrP from cNBH (n = 3), ~52 ± 0.6% from cM1000 at 30% TSD (n = 5), ~60 ± 0.4% at 50% TSD (n = 5), ~58 ± 2% at 70% TSD (n = 5), and ~79 ± 7% at 100% TSD (n = 4; Fig 3A and 3B). In parallel with the ~79% total PrP depletion from cM1000 at 100% of the TSD, ~89 ± 0.3% of at least modestly PK-resistant PrP^Sc^ was also depleted (n = 2; Fig 3C). Importantly, the LTP dysfunction caused by cM1000 preparations from each time point was abolished following the depletion of PrP species (Fig 3D–3G) wherein the LTPs generated by slices treated with dM1000 from 30%, 50%, 70%, and 100% of the TSD (161 ± 7%, 157 ± 6%, 163 ± 5%, and 169 ± 5%, respectively; n = 5 for each) were no longer different from the LTP of slices treated with dNBH (163 ± 6%; n = 5) (Fig 3H). Consistent with the rescue of LTP, the degree of PPF ratio decline was no longer significantly lower in slices treated with dM1000 compared to slices treated with dNBH consistent with rescue of the impairment of the probability of neurotransmitter release during LTP through the immuno-depletion of PrP (Fig 3I). Further, the PTP was no longer disrupted following treatment with dM1000 from 30% and 50% of the TSD (n = 5 each; Fig 3J). In contrast, relative to the dNBH control, the PTP remained impaired after treatment with dM1000 from 70% (*p = 0*.*0221*; n = 5) and 100% (*p = 0*.*0009*; n = 5) of the TSD regardless of the significant rescue of LTP by the immuno-depletion of PrP species (Fig 3J). This inconsistent rescue of PTP aligned with the significant rescue of RRP replenishment after depletion of PrP when treated with dM1000 from 30% and 50% of the TSD (significant increase of RRP size at train 2 and again at train 3), while it remained impaired by dM1000 from 70% and 100% of the TSD (failure of RRP size to significantly increase from train 2 to train 3) (S3M–S3P Fig). Both the probability of release and RRP depletion during HFS trains were normal in slices treated with dM1000 similar to those treated with dNBH (S3D, S3H–S3J Fig). Importantly, dNBH negative controls did not affect synaptic functions as compared to aCSF technical controls (S3A–S3G, S3K & S3L).

### Acute synaptotoxicity at earlier time points is associated with PK-sensitive PrP species

**Fig 4:**
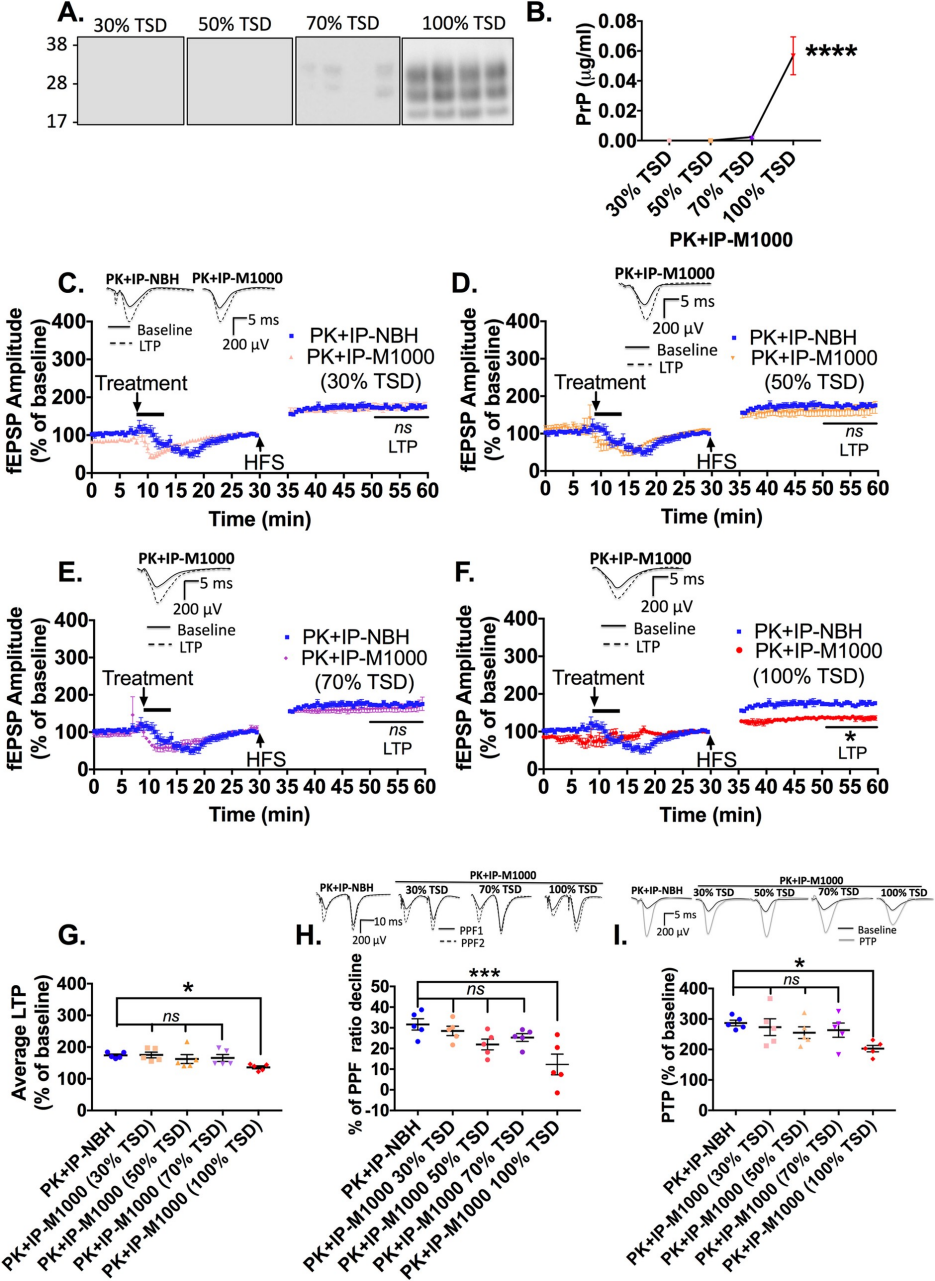
Acute synaptotoxicity at earlier time points of M1000 prion disease is associated with PK-sensitive prion protein species. (A) Western blotting of 20μL of preparations obtained following immuno-precipitation of total PrP species from 1% (w/v) crude M1000 brain homogenates (cM1000) from 30%, 50%, 70%, and 100% of M1000 disease progression to the terminal stage of disease (TSD; in WT C57BL6J mice) and mild PK digestion (probed with 8H4; molecular weight markers are provided at left). (B) Quantification of at least modestly PK-resistant PrP species obtained in A (by densitometry analysis) with the levels of PrP in each PK-eluted immuno-precipitated M1000 preparation (PK+IP-M1000) compared across the time points (One-way ANOVA with Tukey’s correction for multiple comparisons). (C to F) LTP of WT mouse hippocampal slices following a five minute treatment with 0.5% (w/v in aCSF) PK+IP-M1000 from 30% (C), 50% (D), 70% (E), and 100% (F) of the TSD compared with LTP of slices treated with 0.5% (w/v in aCSF) PK-eluted immuno-precipitated normal brain homogenate (PK+IP-NBH) controls (One-way ANOVA with Dunnett’s correction for multiple comparisons). (G) The average LTPs, (H) the average percentage of PPF ratio decline, and (I) the average PTPs generated by WT mouse hippocampal slices treated with PK+IP-NBH and PK+IP-M1000 from across time points of the disease progression (compared by One-way ANOVA with Dunnett’s correction for multiple comparisons). (C-F) The 5 minute treatment started after 8–to-10 minutes of stable baseline. The high frequency stimulation (HFS) trains were applied following 30 minutes of baseline recordings. Results are presented as mean ± standard error of mean. (C-F, H & I) Some examples of raw fEPSP traces are provided as insets. *p<0.*05*, ****p<0.*0001*, *ns =* not statistically significant (*p>0*.*05*).

In a previous report, we described that the prion acute synaptotoxicity harboured in brains at the terminal stage of M1000 prion disease appeared directly linked to at least modestly PK-resistant PrP species [31]; however, the virtual absence of at least modestly PK-resistant PrP species at 30% and 50% of the TSD (Fig 1A–1C and S1A Fig) regardless of the link between total PrPs and acute synaptotoxicity suggests that synaptotoxic species at earlier time points probably relate to PK-sensitive PrP^Sc^. To verify this speculation, we immuno-precipitated total PrP species from cM1000 at each time point, digested pellets with the same mild PK treatment to elute immuno-captured PrP species, and assessed their toxicity using our electrophysiology paradigm. Using routine western blotting, there was no detectable PK-resistant PrP^Sc^ in immuno-captured PrP species prepared from 30% and 50% of the TSD, while very minimal levels were detected at 70% of the TSD (<<0.012μg/mL; n = 5) and substantial amounts at 100% of the TSD (~0.057 ± 12 μg/mL; n = 4; *p<0*.*0001*; One-way ANOVA with Tukey’s correction for multiple comparisons) (Fig 4A and 4B). Importantly, the LTP was not impaired by treatment with PK+IP-M1000 from 30%, 50% and 70% of the TSD relative to the PK+IP-NBH controls (Fig 4C–4E; n = 5 for each), while congruent with our previous observations the LTP was significantly disrupted by exposure to PK+IP-M1000 from 100% of the TSD (Fig 4F, *p = 0*.*0176*; n = 5 each; [31]). Overall, similar to the results in cM1000 (Fig 2I), there was a correlation between disease progression and LTP dysfunction due to the presence of at least modestly PK-resistant PrP^Sc^ largely driven by the results at 100% of the TSD (Fig 4G, *p = 0*.*0264*; One-way ANOVA with Dunnett’s correction for multiple comparisons). Additionally, relative to the normal PPF ratio decline in slices treated with PK+IP-NBH control, levels of PPF ratio decline in slices treated with PK+IP-M1000 from 30%, 50%, and 70% of the TSD also appeared normal (Fig 4H), while the degree of PPF ratio decline in slices treated with PK+IP-M1000 from 100% of the TSD was still significantly lower (Fig 4H; *p = 0*.*0011*: One-way ANOVA with Dunnett’s correction for multiple comparisons). These PPF ratio results revealed that synaptotoxic PrP^Sc^ species responsible for the disruption of the probability of neurotransmitter release during LTP expression are highly PK-sensitive at earlier stages of the disease evolution and acquire resistance to PK as part of biochemical maturation toward the terminal stage of the disease. Parallel results were obtained with PTP dysfunction wherein only the PTP of slices treated with the PK+IP-M1000 from 100% of the TSD were impaired (*p = 0*.*0222*), but not with the PK+IP-M1000 from earlier time points (Fig 4I; One-way ANOVA with Dunnett’s correction for multiple comparisons). The PK+IP-NBH did not cause any background disruption of LTP, PTP, and PPF ratios relative to aCSF technical controls (S4A–S4C Fig).

### Propagation of oligomeric PrP^Sc^ species correlates with the detection of prion acute synaptotoxic PrP^Sc^ during the evolution of M1000 prion disease

**Fig 5:**
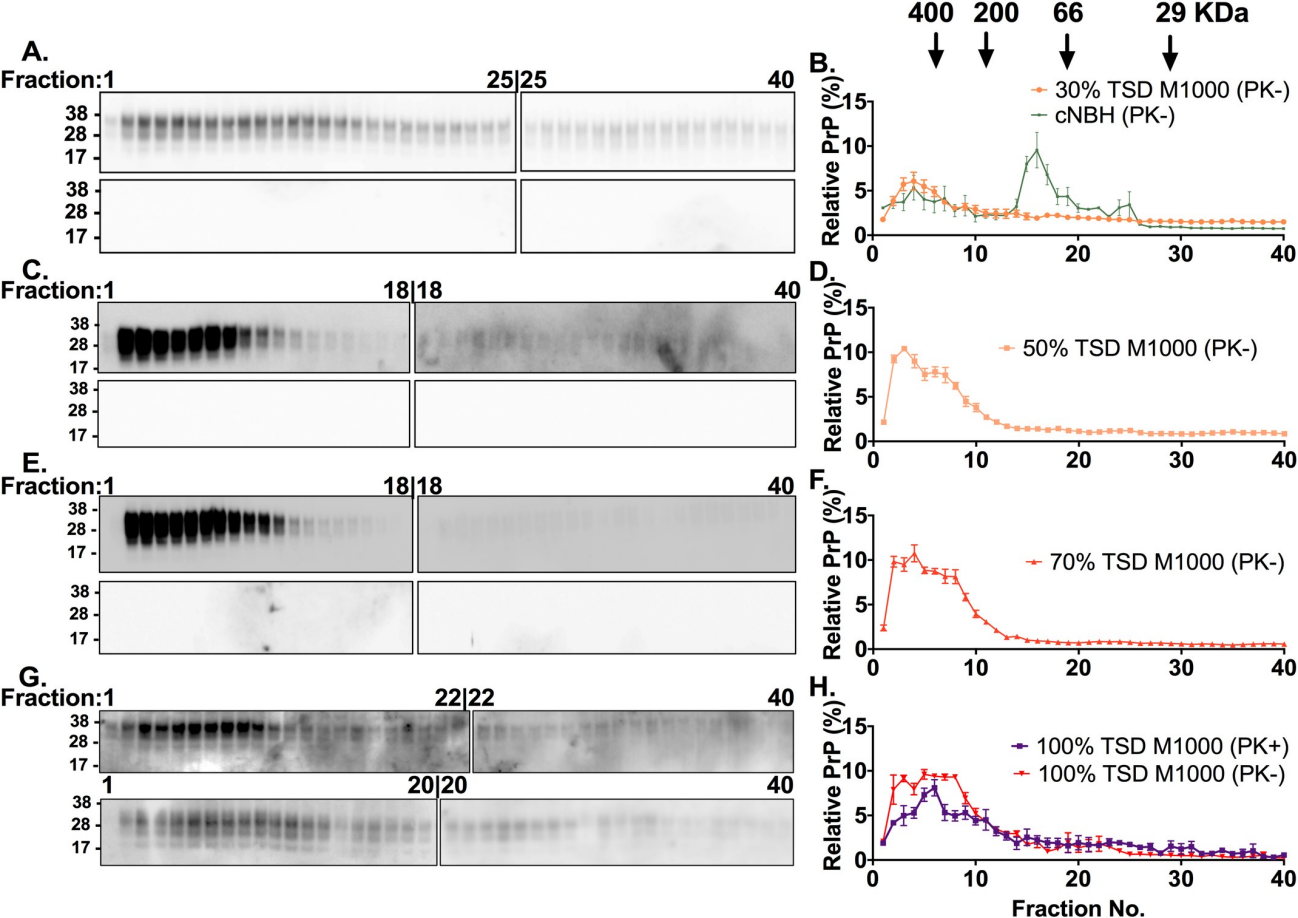
Size fractionation chromatography of M1000 brain homogenates prepared from different time points of the disease progression to the terminal stage. (A, C, E, G) Representative anti-PrP immunoblots of fractions collected after size exclusion chromatography and solubilization of 10% (w/v) crude M1000 brain homogenates from 30% (A), 50% (C), 70% (E), and 100% of the terminal stage of disease (G). The fractions were immunoblotted before (top panel) and after (bottom panel) mild proteinase K treatment (PK; 5μg/mL incubated at 37°C for an hour). Since all 40 fractions could not be loaded in one 26-well gel, two 26-well gels were used for the immunoblotting of fractions from one size exclusion experiment wherein the last fraction on the first gel was also loaded as the first lane on the second gel, thereby allowing the levels of PrP on the second blot to be normalized to those on the first blot. The PrP species were probed by 03R19 antibody. Molecular weight markers are provided at left. (B, D, F) Densitometric analysis of total PrP in fractions from 30% (B; n = 3), 50% (D; n = 3), 70% (F; n = 3) of the terminal stage of disease. (H) Densitometric analysis of total PrP (PK-) and PrP^Sc^ (PK+) in fractions from the terminal stage of the disease (100%; n = 3). (B) Densitometric analysis of the immunoblots of fractions from NBH (data were extracted from our previous publication Fig 3A [31]) is superimposed over the results of M1000 fractions from 30% of the TSD to enhance comparison of PrP species in sham-inoculated mice to those at different stages of prion disease. All data are displayed as mean ± SEM.

Our previous studies demonstrated that acutely synaptotoxic species at the TSD in M1000 prion infection were strongly correlated with at least modestly PK resistant and oligomeric PrP^Sc^ [31]. To further explore the biophysical status of PK-sensitive, acutely synaptotoxic PrP^Sc^ species from earlier time points of M1000 prion disease we undertook size exclusion chromatography to fractionate *ex vivo* preparations from PrP^Sc^ from brains at 30%, 50%, and 70% of the TSD and compared them with *ex vivo* PrP^Sc^ fractions prepared from 100% of the TSD (positive control), as well as PrP^C^ fractions prepared from sham-infected NBH controls (negative control). Our results revealed that oligomeric PrP^Sc^ species were present and predominant at all the time points of the evolution of M1000 prion disease (Fig 5), which appeared directly correlated with the detection of the acutely synaptotoxic species at these earlier stages of the disease. Relative to fractions of the sham-infected NBH controls wherein PrP^C^ species were mostly monomeric (<100kDa in fractions 15 to 40; Fig 5 B), PrP^Sc^ fractions of M1000 preparations were mostly oligomers (>100KDa) wherein most PrP^Sc^ species were eluted in fractions 1 to 10 (Fig 5B, 5D, 5F & 5H). In fact, the most noteworthy change at 30% of the TSD is the loss of predominance of monomeric PrP species, with the predominance of oligomers appearing to be maximised by 50% of the TSD (Fig 5B & 5D). No PK-resistant PrP^Sc^ was detected in the fractions from 30% to 70% of the TSD employing routine western blotting with only fractions from 100% TSD containing at least modestly PK-resistant PrP^Sc^ species (Fig 5A, 5C, 5E & 5G lower panels).

### Oligomeric PrP^Sc^ species propagated during M1000 prion disease progression are acutely synaptotoxic to LTP

**Fig 6:**
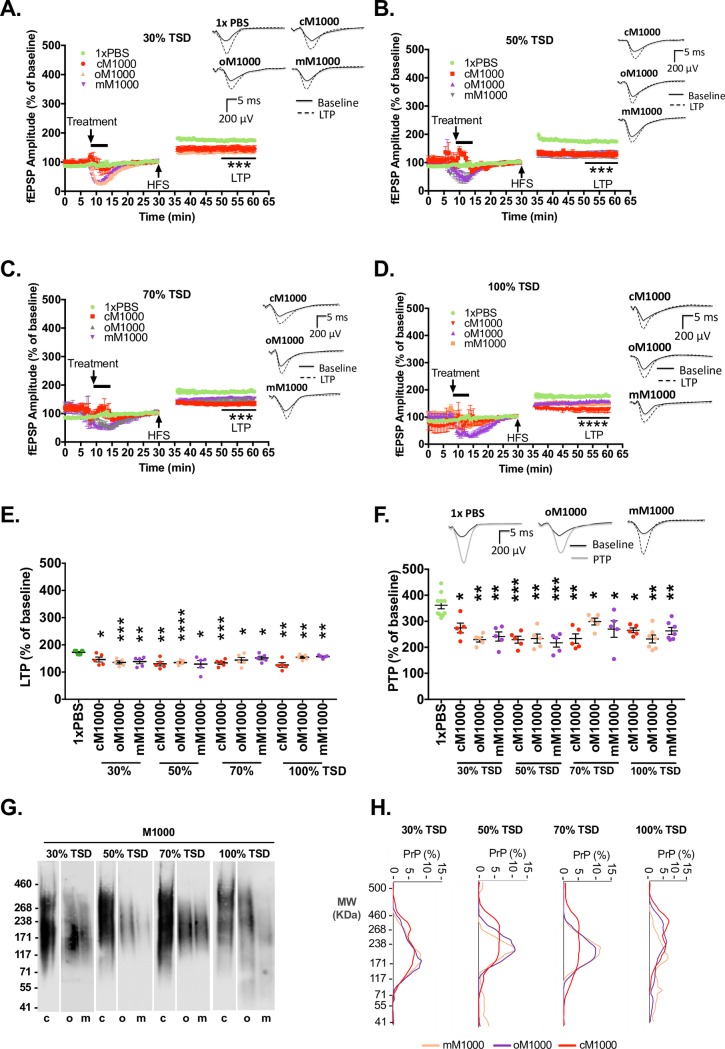
Acute synaptotoxicity of pooled oligomeric and monomeric PrP^Sc^ species. (A-D) LTP from WT hippocampal slices induced by high frequency stimulation (HFS) following treatment for 5-minutes with 1xPBS negative control and M1000 preparations from 30% (A), 50% (B), 70% (C), and 100% (D) of the TSD. These M1000 preparations include crude M1000 brain homogenates (cM1000 positive controls) and pooled M1000 fractions containing either predominantly PrP oligomers (oM1000) or PrP monomers (mM1000). The last 10-minutes of LTP recording of slices treated with M1000 preparations were compared to the LTP of slices treated with 1xPBS (by One-way ANOVA with Dunnett’s correction for multiple comparisons). (E) The average LTPs and average PTPs (F) generated by WT mouse hippocampal slices treated with cM1000, oM1000, and mM1000 from across time points of the disease progression were compared to 1x PBS controls (by One-way ANOVA with Dunnett’s correction for multiple comparisons). (G) Native western blotting of M1000 preparations (20uL in 2% w/v Sarkosyl) used for treatments of WT slices in A-D revealing that monomeric PrP^Sc^ fractionated by size exclusion chromatography appeared to have spontaneously become oligomers. Molecular weight markers are provided at left. (H) Densitometry analysis of blots in G showing that most of the PrP^Sc^ species are oligomers with molecular weights (MW) of at least 150kDa. The 5-minute treatment started after 8–to-10 minutes of stable baseline. The high frequency stimulation (HFS) trains were applied following 30 minutes of baseline recordings. Results are presented as mean ± standard error of mean. (A-D & F) Some examples of raw fEPSP traces are provided as insets. *p<0.*05*, **p<0.*01*, ***p<0.*001*, ****p<0.*0001*, *ns =* not statistically significant (*p>0*.*05*).

To assess the acute synaptotoxicity of different sized assemblies of PrP^Sc^ (predominantly monomeric versus mainly oligomeric) at different time-points of the disease progression, we pooled fractions 1 through 10 to generate oligomer enriched fractions (oM1000) and fractions 15 through 40 to create principally monomeric fractions (mM1000) and assessed their acute synaptotoxicity on WT hippocampal slices. Similar fractions of NBH pooled together as oligomeric NBH (oNBH) and monomeric NBH (mNBH) were assessed for non-specific or background toxicity of brain homogenates introduced through size exclusion chromatography. Crude M1000 brain homogenates (~0.5% w/v) from 100% of the TSD, and crude normal brain homogenates (cNBH, ~0.5% w/v), both processed as for size exclusion chromatography (i.e. Sarkosyl solubilization and exhaustive dialysis), were also used as positive and negative controls for prion acute synaptotoxicity. PBS (1x) alone served as an additional technical negative control for all fractions because it was the size exclusion chromatography buffer, as well as the diluent for PrP fractions. Importantly, cNBH was not toxic to synaptic functions relative to 1x PBS controls (S5A–S5C Fig). In addition, one-to-one dilution of oNBH and mNBH with 1xPBS did not cause any hippocampal synaptic disruption relative to 1xPBS controls (S5A–S5C Fig). In contrast, however, one-to-one dilution of oM1000 from all time points appeared to cause significant impairment of LTP (Fig 6A–6E) and PTP (Fig 6F). Unexpectedly, one-to-one dilution of mM1000 from all the time points was also significantly synaptotoxic to LTP (Fig 6A–6E) and PTP (Fig 6F) relative to 1xPBS controls (compared by One-way ANOVA with Dunnett’s correction for multiple comparisons).

Given evidence that PrP^Sc^ exists in dynamic equilibria [38, 39] with the possibility that monomers separated from oligomers by size exclusion chromatography might rapidly oligomerise in a physiological buffer, we checked if the pooled monomeric fractions prepared for the toxicity assay remained monomers as assessed by native gel western blotting. We found that monomers separated by size exclusion chromatography spontaneously oligomerised (to a size approximating those observed in mM1000 pooled fractions) prior to assessing their acute synaptotoxicity (Fig 6G & 6H), providing a plausible explanation for why pooled mM1000 preparations were also acutely synaptotoxic.

### Propagation of transmitting M1000 prions and acutely synaptotoxic prions display broadly similar temporal profiles

**Fig 7:**
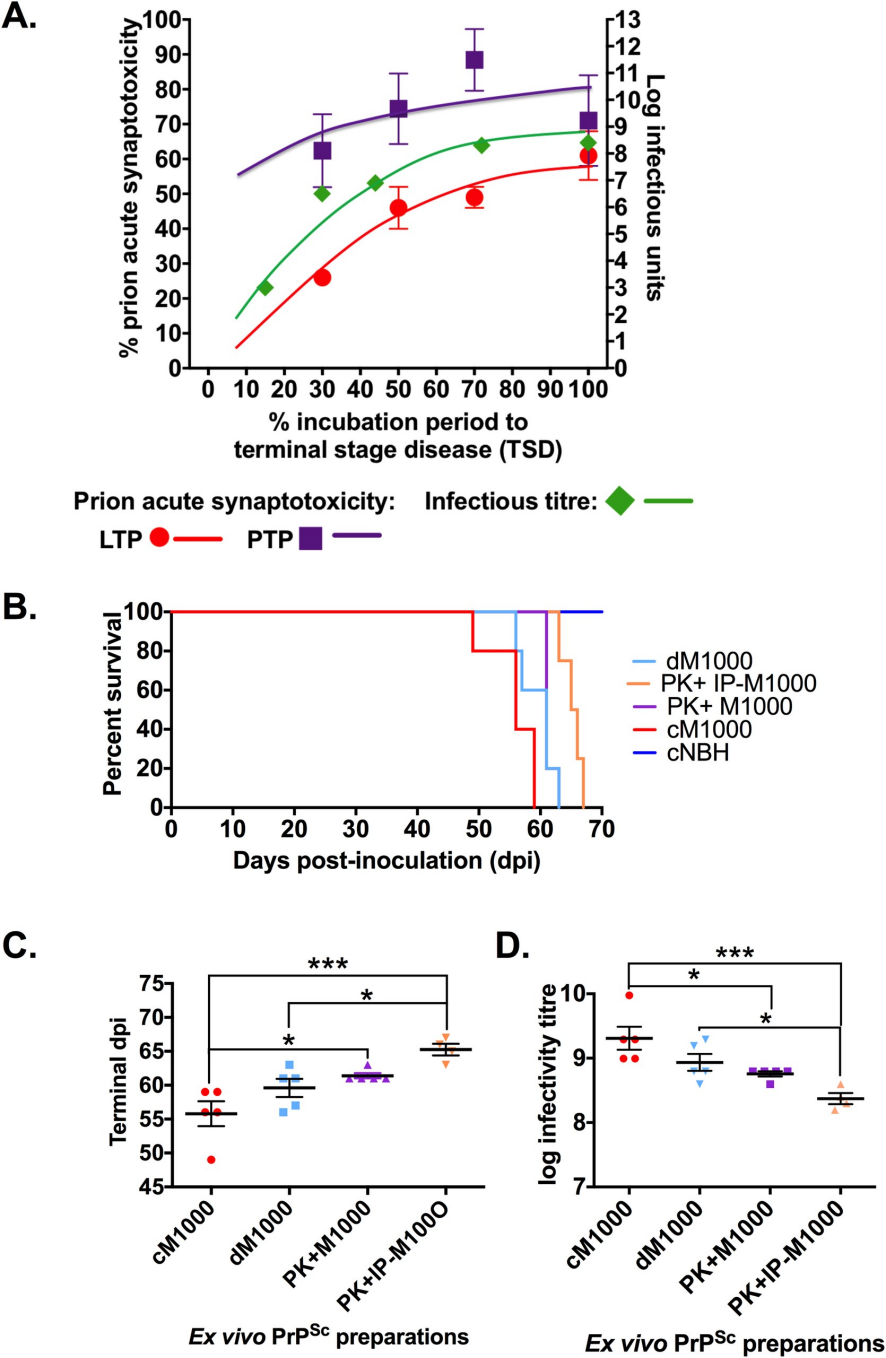
Evidence for efficiently transmitting minimally synaptotoxic PrP^Sc^ species co-existing with separate primarily synaptotoxic species at terminal disease stage. (A) Concomitant representation of the degree of prion acute synaptotoxicity across the disease progression calculated as the percentage of LTP and PTP decline from normal LTP together with titre of infectivity (log ID_50_ units/g brain) across the designated time points as published previously [8]. (B) Kaplan-Meier survival curves of tga20 mice intracerebrally (ic) inoculated with various 1% (w/v) preparations generated from brains of terminally sick M1000 inoculated WT mice, including crude M1000 brain homogenates (cM1000), PrP-immuno-depleted cM1000 (dM1000), 5μg/ml proteinase-K (PK) digested cM1000 (PK+M1000), and reconstituted PK-eluted immuno-precipitated PrP (PK+IP-M1000), as well as tga20 mice sham inoculated with crude NBH (cNBH). (C) Comparison of the average survival (day post-inoculation; dpi) to the TSD in tga20 mice ic inoculated with cM1000, dM1000, PK+M1000, and PK+IP-M1000 (One-way ANOVA with Tukey’s correction for multiple comparisons). (D) Comparison of the estimated mean infectivity titres (log_10_ ID_50_ units/g brain) of the cM1000, dM1000, PK+M1000, and PK+IP-M1000 preparations (One-way ANOVA with Tukey’s correction for multiple comparisons). The mean time to the TSD was used to calculate the approximate infectivity titre of each M1000 preparation using a linear regression formula obtained from previous quantal dose titration of M1000 prion infectivity in tga20 mice [8]. Data are presented as mean ± standard error of mean. *p<0.*05*, ***p<0.*001*.

Despite evidence clearly supporting that PrP^Sc^ is responsible for disease transmission [8, 13] as well as neurotoxicity in prion disease, there has been relatively limited exploration of the propagation profiles of neurotoxic species compared with transmitting species [13, 21]. As described above, the degree of synaptotoxicity in the form of LTP dysfunction across M1000 disease evolution progressively and significantly increased (Fig 2I lower panel; One-way ANOVA with Tukey’s correction for multiple comparisons; *p<0*.*0001*), whereas PTP impairment did not significantly increase across the time points, which may relate to a “ceiling effect” due to the apparent heightened pre-synaptic sensitivity to prion toxicity [31] (Fig 2I upper panel; One-way ANOVA with Tukey’s correction for multiple comparison; *p = 0*.*3306*). To qualitatively compare the propagation of acutely synaptotoxic PrP^Sc^ species to that of transmitting species, we utilised our previous study of the temporal profile of M1000 infectivity in WT mice following ic inoculation [8]. The infectivity titre (log ID_50_ units per μL of 1% [w/v] M1000 brain homogenate) progressively increased from ~6.5 at ~30% of the TSD (~42 dpi) to ~6.9 at ~44% (~64 dpi) of the TSD to plateau at ~8.3 at ~72% of the TSD (~104 dpi). Hence, when plotted for illustrative purposes (Fig 7A), there appeared to be broadly similar propagation profiles for transmitting and acutely synaptotoxic species albeit with plateauing of infectivity noteworthy from ~72% of TSD while synaptotoxic species appear to continue to modestly increase until terminal disease. Further, despite the degree of PTP impairment not being significantly increased across the disease evolution, the overall profile of PTP dysfunction (as illustrated by the best-fit curve) during the disease progression appeared similar to that of LTP dysfunction but at a higher level (Fig 7A).

### Efficiently transmitting minimally synaptotoxic PrP^Sc^ species co-exist with primarily acutely synaptotoxic PrP^Sc^ species at terminal disease stage

We previously showed that cM1000 and modestly PK-treated cM1000 prepared from brains at 100% of the TSD were equivalently synaptotoxic to WT hippocampal CA1 region LTP [31]. This acute synaptotoxicity was demonstrated to be directly associated with PrP species present in cM1000 given the significant rescue of LTP impairment by selective immuno-depletion of ~79% total PrP from cM1000 (Fig 3A; including ~89% of at least modestly PK resistant PrP^Sc^ [Fig 3C] and ~96% of highly protease-resistant PrP^Sc^ (resistant to 50μg/mL for one hour at 37°C) (refer to Fig 2A–2C in [31]) through PrP immuno-precipitation. The direct contribution of PrP^Sc^ to this acute synaptotoxicity was further supported by the return of LTP impairment following exposure of WT hippocampal slices to reconstituted, immuno-precipitated PrP^Sc^ species eluted from pellets by modest PK treatment (Fig 4F and also refer to Fig 2G–2I in [31]).

To determine the infectivity of these various M1000 preparations (cM1000, PK+M1000, dM1000 and PK+IP-M1000) at 100% of the TSD, we undertook incubation time interval bioassays employing routine ic inoculation of tga20 mice, euthanized once they reached the TSD as reflected by reduced spontaneous activity, severe ataxia and hind limb paresis. Control tga20 mice ic inoculated with cNBH did not develop features of prion disease by the time the experiment was terminated (120 dpi). In contrast, the mean time to the TSD of mice inoculated with dM1000 (60 ± 1.7 dpi) was similar to those inoculated with cM1000 (56 ± 1.8 dpi; *p = 0*.*1737*), while modestly but significantly shorter than those inoculated with PK+IP-M1000 (65 ± 1.3; *p = 0*.*0360*; Fig 7B & 7C). The mean time to the TSD of those mice inoculated with PK+M1000 (61 ± 0.4 dpi) was not different from those infected with dM1000 (*p = 0*.*7335*), as well as PK+IP-M1000 (*p = 0*.*2034*) but significantly albeit minimally longer than those infected with cM1000 (*p* = *0*.*0266*; Fig 7B & 7C). The mean time to the TSD was used to calculate the approximate infectivity titre of each M1000 preparation using a linear regression formula (*y* = -11.02*x* + 111.8) obtained from previous quantal dose-titration of M1000 prion infectivity in tga20 mice [8]. The estimated average infectivity titre (log_10_ ID_50_ units/g brain) of dM1000 (~8.9) was not different to cM1000 (~9.2; *p = 0*.*1736*) and PK+M1000 (~8.8; *p = 0*.*7320*) but was significantly although minimally higher than the PK+IP-M1000 (~8.4; *p = 0*.*0343*; Fig 7D). Further, the infectivity titre of the PK+M1000 was significantly lower than the cM1000 (*p* = *0*.*0265*), but not significantly different from the PK+IP-M1000 (*p = 0*.*1961*; Fig 7D). Noteworthy from these collective observations is the substantially abrogated acute synaptotoxicity of dM1000 while retaining essentially unaltered high levels of infectivity (despite removal of ~77% of total PrP species), which stands in contrast to the prominent acute synaptotoxicity of PK+IP-M1000 with its modestly albeit significantly reduced infectivity.

## Discussion

The primary purpose of this body of work was to gain insights into the time of occurrence and biophysical properties of acutely synaptotoxic PrP^Sc^. Employing a combination of experimental approaches the current study has demonstrated some important observations, especially: that acutely synaptotoxic PrP^Sc^ species related to M1000 prions are generated from early in disease evolution generally coinciding with the propagation of transmitting species; and that acutely synaptotoxic PrP^Sc^ species most likely constitute small oligomers, appearing to undergo significant biochemical transformation relatively late in the incubation period, particularly transition from quite protease-sensitive to at least modestly protease-resistant. The early presence of synaptotoxic conformers in combination with the relative stability of their size fractionation profile and only comparatively modest increase in absolute acute synaptotoxicity from 50% of the TSD suggests that progressive failure of neuro-protective mechanisms is likely to be an important component of the eventual transition to overt prion disease.

The ability to detect the presence of acutely synaptotoxic PrP^Sc^ in the brains of M1000 infected mice at 30% of the TSD was independent of the host animal species (Balb/c or C57BL/6J mice) and method of inoculation (routine or stereotaxic ic injection). Moreover, in further contrast to the reported plateauing of the production of infectious PrP^Sc^ in the latter phase of the disease [8, 12, 21], possibly related to declining PrP^C^ levels [13], we found propagation of synaptotoxic PrP^Sc^ appeared to increase throughout disease development although the capacity of cM1000 to induce PTP failure appeared maximal even at the earliest stage of disease progression assessed, in keeping with previous findings suggesting an enhanced pre-synaptic vulnerability [31]. The explanation for discrepancies in the reported time of occurrence of neurotoxic PrP^Sc^ conformers during disease progression is unclear. Some evidence suggests that the prion strain utilised is unlikely to be a major influence [22] underscoring that methodological factors, especially the sensitivity of techniques employed to discern neurotoxicity may be important contributors. Indeed, we have recently argued that somewhat hindering previous efforts to detect neurotoxic prion species present in *ex vivo* preparations from early time points of disease has been the relative lack of tractable, sensitive, detection paradigms [27]. Congruent with this speculation is that *in vivo* studies reporting the late, sequential propagation of neurotoxic PrP^Sc^ species have relied primarily on the occurrence of typical clinical features of murine prion disease [12, 21], while those describing findings supporting an earlier propagation of toxic conformers have utilised techniques such as serial specific electrophysiological or behavioural interrogation across the incubation period [18, 20]. Of particular note, the observation of acutely synaptotoxic PrP^Sc^ at 30% of the TSD aligns with a study reporting progressive failure of hippocampal CA1 LTP from ~44% of the incubation period during ME7 prion infection [18] and also correlates with our previous report that *in vivo* M1000 infection is associated with elevated by-products of lipid peroxidation from ~30% of the TSD with typical neuropathological changes occurring from ~57% of the TSD, well in advance of the plateauing of infectivity at ~72% of the TSD [8]. Evidence also supports that elevated free radicals in neurodegenerative diseases are neurotoxic and can impair synaptic functions [40–42]. Because the levels of free by-products of lipid peroxidation are elevated at earlier stages of M1000 prion disease [8], we cannot exclude that part of the early synaptotoxicity is due to the presence of free radicals related to propagation of PrP^Sc^; however, the abrogation of synaptotoxicity with selective immuno-depletion of PrP coupled to the fact that levels of free lipid peroxidation by-products progressively decline from around 50% of the TSD [8] appears inconsistent with heightened oxidative stress being the primary driver of the progressive increase in the synaptotoxicity across the latter disease progression that we (in this study) and others [18] have observed.

Previously we reported that acutely synaptotoxic PrP^Sc^ at the TSD in M1000 infection were most likely constituted as small oligomers [31]. The present findings elaborate this observation and suggest that acutely synaptotoxic PrP^Sc^ species are oligomeric from inception as well as protease-sensitive, which is in broad agreement with previous suggestions regarding the biophysical nature of neurotoxic species during disease progression [13, 34]. The observed biochemical evolution of such oligomeric PrP^Sc^ relatively late in the incubation period (after 70% of the TSD) from quite protease-sensitive to at least modestly protease-resistant is consistent with a previous report using the same prion strain (Fukuoka-1) [8, 34]. Further illustrating the biochemical transformation of PrP^Sc^ in the latter part of the incubation period and similar to what was reported by Sasaki and colleagues [34] is the apparently altered solubility or interaction of misfolded PrP^Sc^ conformers in NaPTA. Notwithstanding the reduced sensitivity of routine western blot detection, we observed very little PrP^Sc^ after modest PK digestion at 70% of the TSD without NaPTA pre-treatment while substantial quantities were detectable when utilising NaPTA precipitation. Of note though, despite PK-resistant PrP^Sc^ levels being substantially increased at 70% of the TSD as determined by employing NaPTA pre-treatment of cM1000, they do not yet represent the acutely synaptotoxic PrP^Sc^ species given that modest protease treatment is able to completely attenuate the synaptotoxicity of immuno-precipitated PrP^Sc^ derived from this time point. These findings underscore a likely cumulative or step-wise maturation of the biophysical properties of synaptotoxic oligomers with protease-resistance perhaps one of the last hallmark changes to occur.

Acknowledging the reported inherent imprecision of incubation time interval assays (±0.5 log_10_ median infective dose units) when calculating infectivity [3], we believe only estimates outside this range are reliably different. Importantly, our bioassays showed that the estimated infectivity of dM1000 was not clearly different to that of mice inoculated with cM1000, while PK+IP-M1000 was lower in infectivity with respect to cM1000 and probably significantly reduced compared to dM1000. It is noteworthy that although a ~0.8 log_10_ relative decrease in infectivity between cM1000 and PK+IP-M1000 preparations appears rather modest it equates to an absolute reduction of the order of 1,300 million ID_50_ units/g brain. It is also worth emphasising that while depletion of ~79 ± 7% of total PrP to generate dM1000 at 100% of the TSD was sufficient to significantly ameliorate synaptotoxicity, the remaining ~21% harboured infectivity that was not clearly different to that contained in cM1000 brain homogenate. Further, employing the same immuno-depletion method, including anti-PrP capture and detection antibodies, we previously showed that an essentially identical reduction in total PrP species of ~77 ± 9% from cM1000 at 100% of the TSD was associated with ~96 ± 4% reduction in highly protease-resistant PrP^Sc^ [31] in addition to the ~89% reduction of at least modestly PK-resistant species we now report, with reciprocal enrichment of PK-resistant PrP^Sc^ confirmed in PK+IP-M1000 preparations. Consequently, the unaltered infectivity in the presence of substantial depletion of PK-resistant PrP^Sc^ in dM1000 supports that the residual predominantly PK-sensitive conformers are highly infectious species.

We previously attributed the ongoing impairment of PTP by dM1000 at 100% of the TSD to an enhanced pre-synaptic vulnerability to the small residual amount of PK-resistant PrP^Sc^ [31]; however, some refinement of this simple explanation appears necessary when trying to encompass results at earlier time points. At 70% of the TSD, despite similar absolute amounts of total PrP remaining after immuno-depletion when compared to 30% and 50% of the TSD, only dM1000 from 70% caused PTP impairment, with modest PK treatment of the complementary 60% of total PrP captured in the immuno-precipitated pellets able to completely abrogate any acute synaptotoxicity. Although alternative explanations cannot be excluded, these data are compatible with a differential interaction of the immuno-capturing antibody with PK-sensitive synaptotoxic PrP^Sc^ at 70% of the TSD stemming from its evolving biochemical transformation (akin to the differential interaction of PrP^Sc^ with NaPTA at this time point), as suggested by previous results we reported (Fig 3 [31], compare panel F with panel H), such that less PTP impairing species are removed by immuno-precipitation leaving sufficient to impair this pre-synaptic function. In addition, we also reported that PK+IP-M1000 preparations at 100% of the TSD contained full acute synaptotoxicity equivalent to or even a little greater than that caused by cM1000 [31]. Collectively we construe these previous and present findings as supporting the likelihood that pathogenic PrP^Sc^ at 100% of the TSD clusters into at least two overlapping but relatively separable biophysical ensembles: one, PrP^Sc^ species that are minimally or non-synaptotoxic but highly efficient in transmission harbouring little PK-resistance; and a second group, highly synaptotoxic species replete in PK-resistant conformers that retain substantial infectivity. Given that the infectivity titres estimated by our incubation time interval approach for all preparations were within a one log_10_ of each other suggests that infectivity is perhaps an integral feature of all PrP^Sc^ species at the TSD.

The observation that acutely synaptotoxic PrP^Sc^ up to and including 70% of the TSD was highly protease-sensitive rendered our experimental approach (PrP immuno-precipitation coupled to elution through modest PK treatment of pellets) impracticable to rigorously assess whether separable pathogenic species could be verified at earlier time points; consequently we restricted these evaluations to 100% of the TSD by which stage acutely synaptotoxic conformers are sufficiently resistant to the modest PK treatment required to elute them from immuno-precipitation pellets to allow their subsequent use in electrophysiology experiments. Also, although size exclusion chromatography profiles appeared generally similar over the disease evolution albeit with an apparent increase in predominance of higher molecular weight fractions between 30% and 50% of the TSD, we did not quantify absolute amounts of PrP^Sc^ species. Hence, this leaves unresolved whether acutely synaptotoxic PrP^Sc^ species, especially PK-sensitive species at earlier time points in disease evolution, may harbour greater synaptotoxicity per notional “toxic unit” and whether the intrinsic synaptotoxicity per “toxic unit” can change over the course of disease evolution. This concept is similar to that of a previous study of the M1000 prion strain utilising subcellular fractions containing PrP^Sc^ prepared from M1000-infected mouse RK13 cells wherein some fractions were shown to harbour equally efficient transmissibility despite much lower levels of PK-resistant PrP^Sc^ [43].

In summary, the current study is the first practical application of our recently developed electrophysiological paradigm designed to assess the presence of acutely synaptotoxic *ex vivo* PrP^Sc^, demonstrating that synaptotoxic species related to M1000 prions are generated from early in disease evolution broadly overlapping the propagation profile of transmissible species. The very short period over which our assay is performed militates against significant propagation of *de nov*o synaptotoxic PrP^Sc^ and also limits the time available for attenuating compensatory or neuroprotective mechanisms thereby enhancing the specificity and sensitivity for detecting directly synaptotoxic species. Conventional indicators of the presence of neurotoxic prion species during disease evolution such as the presence of neuropathological changes or the development of overt clinical signs [12, 27] are arguably less sensitive metrics because they only become manifest when overall or regional adaptive and/or neuroprotective CNS thresholds have been persistently exceeded by accumulating neurotoxic species [28].

## Supporting information

S1 Fig Routine western blotting of crude brain homogenates from M1000-infected mice and sham-infected mice. (A) Western immunoblots of 20 μL crude 1% (w/v) M1000 brain homogenates (cM1000) from 30%, 50%, 70% and 100% of M1000 prion disease progression to the terminal stage disease (TSD) before (-) and after (+) digestion with PK (5μg/mL) at 37°C for an hour (Upper panel: PK-; Lower panel: PK+). PrP species were probed with 8H4 antibody. Total protein was stained with Commassie blue stain (Coom.) as a loading control. (B) Western immunoblots of 20 μL crude 1% (w/v) normal brain homogenates (cNBH) obtained from mice sham inoculated with uninfected brain homogenates and culled at time points equivalent to the 30%, 50%, 70% and 100% of the TSD of M1000 disease showed no change in total PrP or any evidence of PK-resistant PrP. PrP levels before and after PK digestion (5μg/mL at 37°C for an hour) were probed with 03R19 antibody.
(TIF)

S2 Fig Other electrophysiology results related to Fig 2. (A) The average LTPs, (B) the average PTP, and (C) the average percentage of PPF ratio decline generated by slices treated with cNBH compared to aCSF technical controls by unpaired Student’s t-test. (D) Average probability of neurotransmitter release evoked by each of the three HFS trains (T1, T2, & T3; determined by dividing the fEPSP amplitude of pulse 2 by that of pulse 1) were compared within each treatment group by One-way ANOVA with Dunnett’s correction for multiple comparisons. (E) Readily releasable pool (RRP) depletion during (E) T1, (F) T2, and (G) T3 in slices treated with cNBH compared to aCSF controls by one phase decay exponential function (comparing the time-constant of fEPSP amplitude decay from pulse 3 to pulse 9). RRP depletion during (H) T1, (I) T2, and (J) T3 in slices treated with cNBH compared to cM1000 from across four time-points of the disease progression. Replenishment of RRP following each train of the three HFS trains was measured in slices treated with (K) aCSF controls, (L) cNBH, and cM1000 from (M) 30%, (N) 50%, (O) 70% and (P) 100% of the TSD. See Methods for how the RRP size and RRP replenishment were estimated. Results are presented as mean ± standard error of mean. *p<0.*05*, **p<0.*01*, ***p<0.*001*, *ns =* not statistically significant (*p>0*.*05*).
(TIF)

S3 Fig Other electrophysiology results related to Fig 3. The average LTPs, (B) the average PTP, and (C) the average percentage of PPF ratio decline generated by slices treated with dNBH compared to aCSF technical controls by unpaired Student’s t-test. (D) Average probability of neurotransmitter release evoked by each of the three HFS trains (T1, T2, & T3; determined by dividing the fEPSP amplitude of pulse 2 by that of pulse 1) were compared within each treatment group by One-way ANOVA with Dunnett’s correction for multiple comparisons. (E) Readily releasable pool (RRP) depletion during (E) T1, (F) T2, and (G) T3 in slices treated with cNBH compared to aCSF controls by one phase decay exponential function (comparing the time-constant of fEPSP amplitude decay from pulse 3 to pulse 9; see Methods for details). RRP depletion during (H) T1, (I) T2, and (J) T3 in slices treated with dNBH compared to dM1000 from across four time-points of the disease progression. Replenishment of RRP following each train of the three HFS trains was measured in slices treated with (K) aCSF controls, (L) dNBH, and dM1000 from (M) 30%, (N) 50%, (O) 70% and (P) 100% of the TSD. See Methods for how the RRP size and the RRP replenishment were estimated. Results are presented as mean ± standard error of mean. *p<0.*05*, **p<0.*01*, ***p<0.*001*, *ns =* not statistically significant (*p>0*.*05*).
(TIF)

S4 Fig Other electrophysiology results related to Fig 4. The average LTPs, (B) the average PTPs, and (C) the average percentages of PPF ratio decline generated by slices treated with dNBH compared to aCSF technical controls by unpaired Student’s t-test. Results are presented as mean ± standard error of mean. *ns =* not statistically significant (*p>0*.*05*).
(TIF)

S5 Fig Electrophysiology results related to pooled size fractionated M1000 and normal brain homogenates displayed in main Fig 6. (A) LTP of WT mouse hippocampal slices following a five-minute treatment with 1x PBS negative technical control compared to ~0.5% (w/v in 1x PBS) cNBH after processing for size exclusion chromatography, as well as pooled oligomeric and monomeric fractions of NBH after size fractionation in 1x PBS. The five-minute treatment started after eight to 10 minutes of stable baseline. The high frequency stimulation (HFS) trains were applied following 30 minutes of baseline recordings. The average LTPs (B) and average PTPs (C) of slices treated with cNBH, oNBH, and mNBH were compared to those of slices treated with 1x PBS by One-way ANOVA with Dunnett’s correction for multiple comparisons. Results are presented as mean ± standard error of mean. *ns* = not statistically significant (*p>0*.*05*).
(TIF)
