# Supplementary figures and images for: Early existence and biochemical evolution characterise acutely synaptotoxic PrPSc
Source: PLoS Pathog. 2019 Apr 10;15(4):e1007712. doi: 10.1371/journal.ppat.1007712 (PMC6490942; doi:10.1371/journal.ppat.1007712)

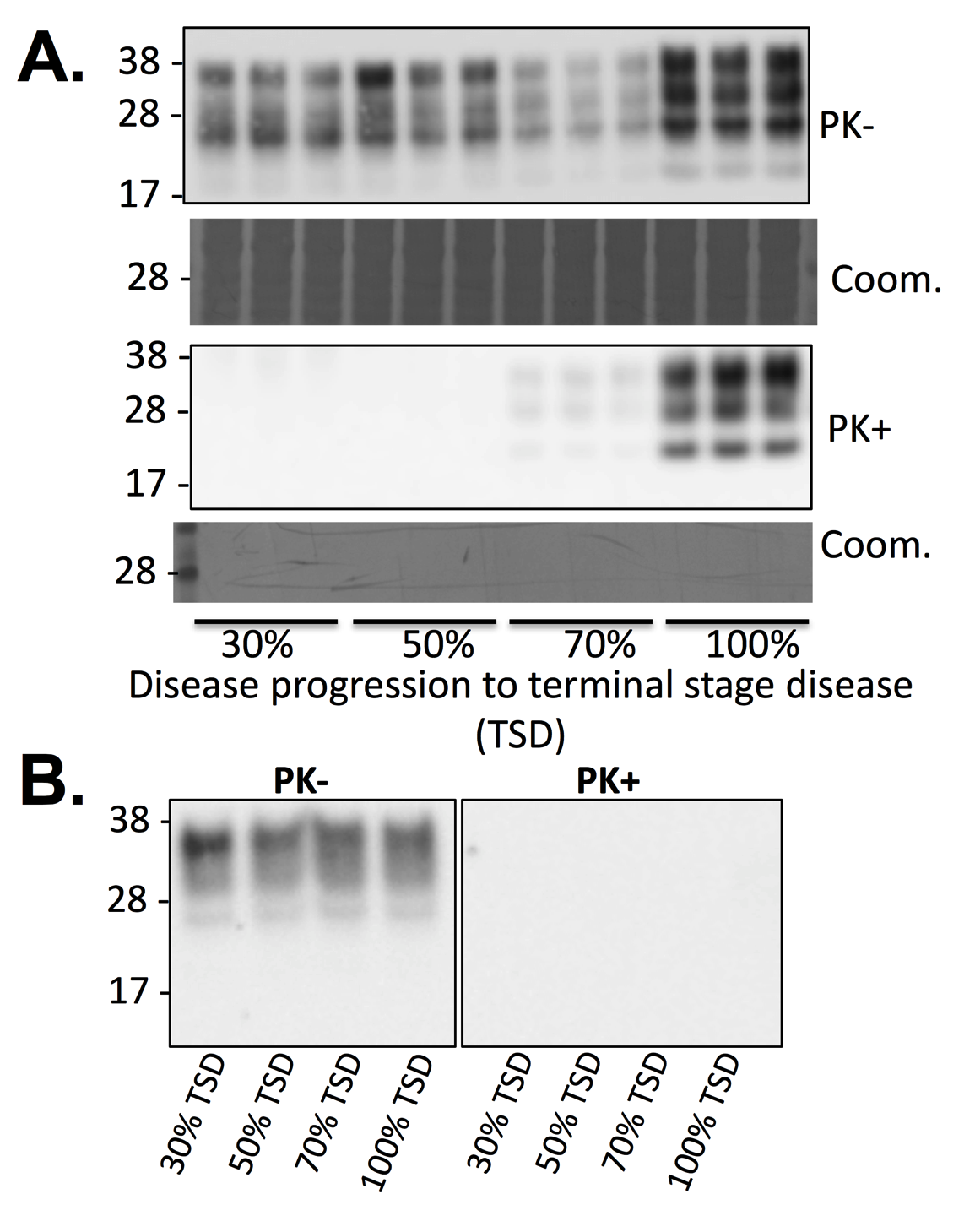

Supplement: S1 Fig — (A) Western immunoblots of 20 μL crude 1% (w/v) M1000 brain homogenates (cM1000) from 30%, 50%, 70% and 100% of M1000 prion disease progression to the terminal stage disease (TSD) before (-) and after (+) digestion with PK (5μg/mL) at 37°C for an hour (Upper panel: PK-; Lower panel: PK+). PrP species were probed with 8H4 antibody. Total protein was stained with Commassie blue stain (Coom.) as a loading control. (B) Western immunoblots of 20 μL crude 1% (w/v) normal brain homogenates (cNBH) obtained from mice sham inoculated with uninfected brain homogenates and culled at time points equivalent to the 30%, 50%, 70% and 100% of the TSD of M1000 disease showed no change in total PrP or any evidence of PK-resistant PrP. PrP levels before and after PK digestion (5μg/mL at 37°C for an hour) were probed with 03R19 antibody. (TIF) [file ppat.1007712.s001.tif]

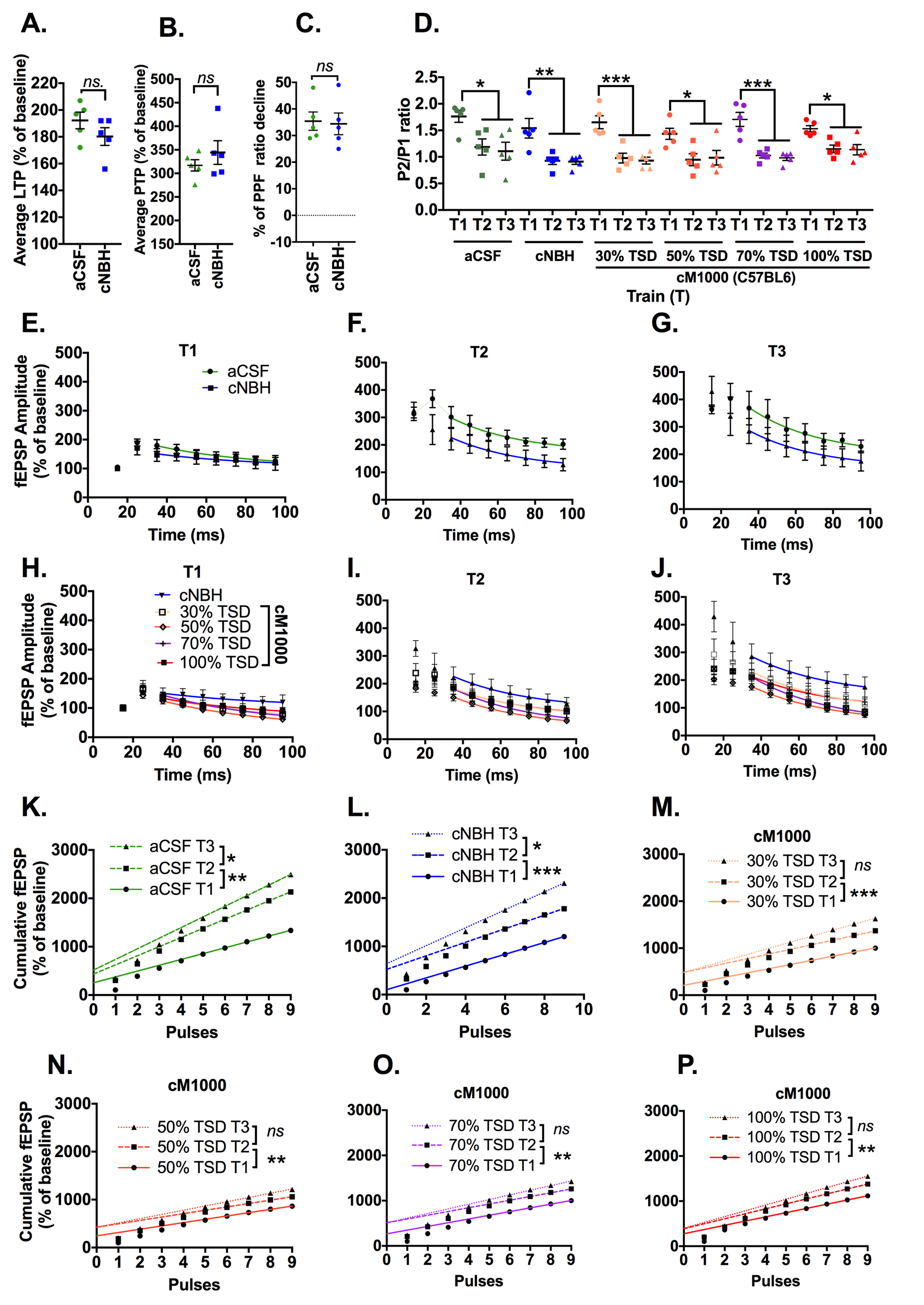

Supplement: S2 Fig — (A) The average LTPs, (B) the average PTP, and (C) the average percentage of PPF ratio decline generated by slices treated with cNBH compared to aCSF technical controls by unpaired Student’s t-test. (D) Average probability of neurotransmitter release evoked by each of the three HFS trains (T1, T2, & T3; determined by dividing the fEPSP amplitude of pulse 2 by that of pulse 1) were compared within each treatment group by One-way ANOVA with Dunnett’s correction for multiple comparisons. (E) Readily releasable pool (RRP) depletion during (E) T1, (F) T2, and (G) T3 in slices treated with cNBH compared to aCSF controls by one phase decay exponential function (comparing the time-constant of fEPSP amplitude decay from pulse 3 to pulse 9). RRP depletion during (H) T1, (I) T2, and (J) T3 in slices treated with cNBH compared to cM1000 from across four time-points of the disease progression. Replenishment of RRP following each train of the three HFS trains was measured in slices treated with (K) aCSF controls, (L) cNBH, and cM1000 from (M) 30%, (N) 50%, (O) 70% and (P) 100% of the TSD. See Methods for how the RRP size and RRP replenishment were estimated. Results are presented as mean ± standard error of mean. *p<0.05, **p<0.01, ***p<0.001, ns = not statistically significant (p>0.05). (TIF) [file ppat.1007712.s002.tif]

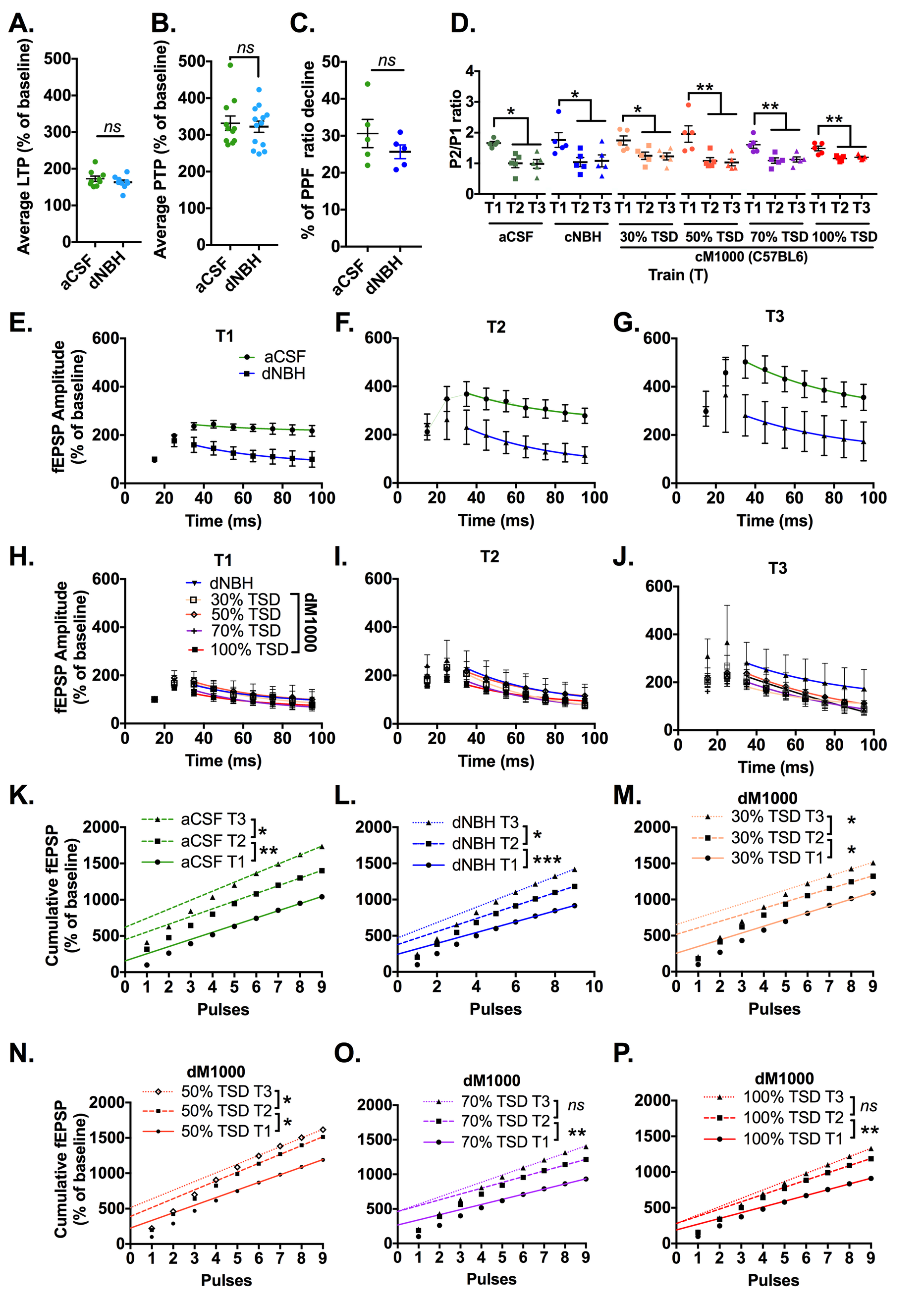

Supplement: S3 Fig — The average LTPs, (B) the average PTP, and (C) the average percentage of PPF ratio decline generated by slices treated with dNBH compared to aCSF technical controls by unpaired Student’s t-test. (D) Average probability of neurotransmitter release evoked by each of the three HFS trains (T1, T2, & T3; determined by dividing the fEPSP amplitude of pulse 2 by that of pulse 1) were compared within each treatment group by One-way ANOVA with Dunnett’s correction for multiple comparisons. (E) Readily releasable pool (RRP) depletion during (E) T1, (F) T2, and (G) T3 in slices treated with cNBH compared to aCSF controls by one phase decay exponential function (comparing the time-constant of fEPSP amplitude decay from pulse 3 to pulse 9; see Methods for details). RRP depletion during (H) T1, (I) T2, and (J) T3 in slices treated with dNBH compared to dM1000 from across four time-points of the disease progression. Replenishment of RRP following each train of the three HFS trains was measured in slices treated with (K) aCSF controls, (L) dNBH, and dM1000 from (M) 30%, (N) 50%, (O) 70% and (P) 100% of the TSD. See Methods for how the RRP size and the RRP replenishment were estimated. Results are presented as mean ± standard error of mean. *p<0.05, **p<0.01, ***p<0.001, ns = not statistically significant (p>0.05). (TIF) [file ppat.1007712.s003.tif]

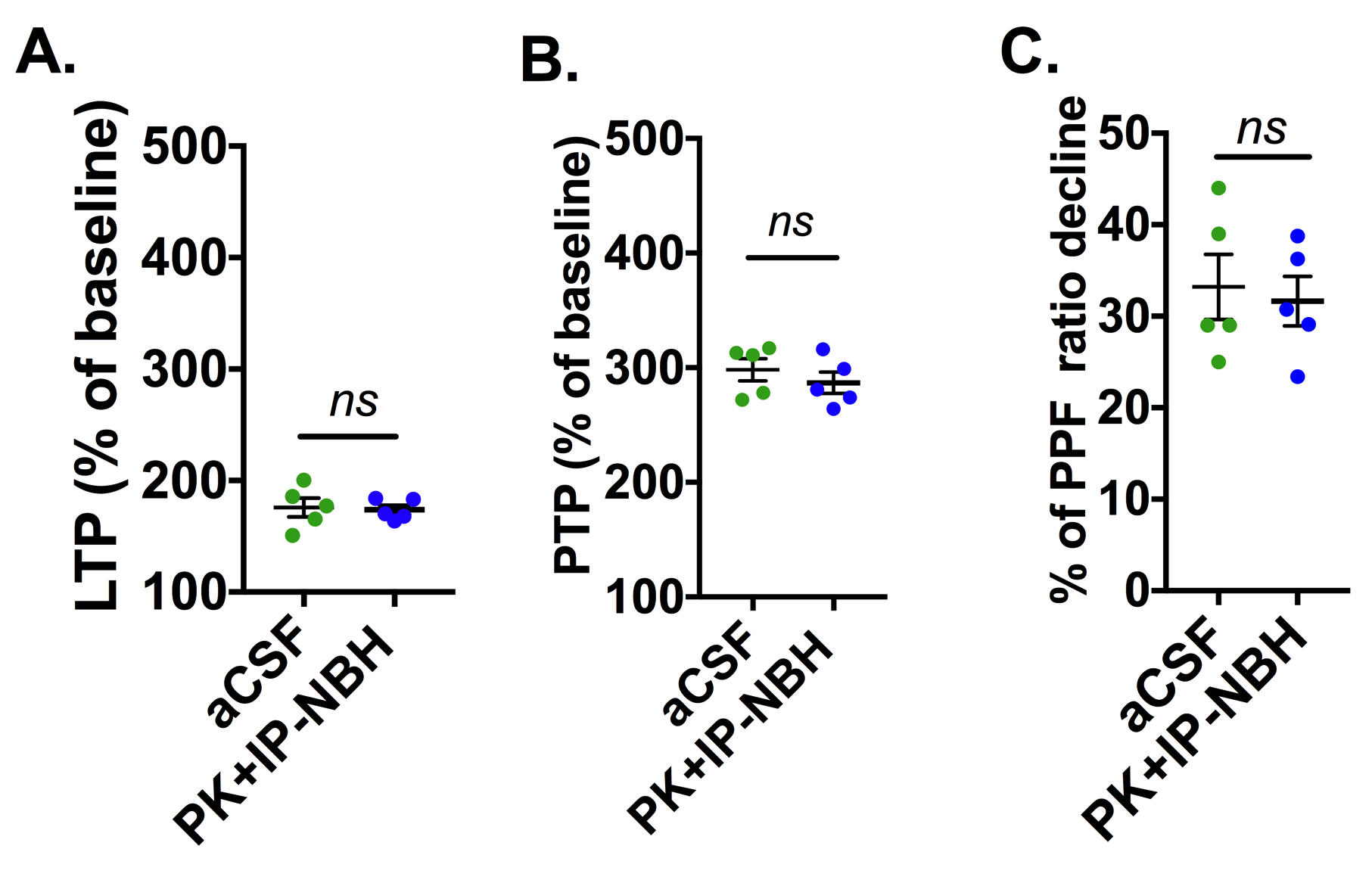

Supplement: S4 Fig — The average LTPs, (B) the average PTPs, and (C) the average percentages of PPF ratio decline generated by slices treated with dNBH compared to aCSF technical controls by unpaired Student’s t-test. Results are presented as mean ± standard error of mean. ns = not statistically significant (p>0.05). (TIF) [file ppat.1007712.s004.tif]

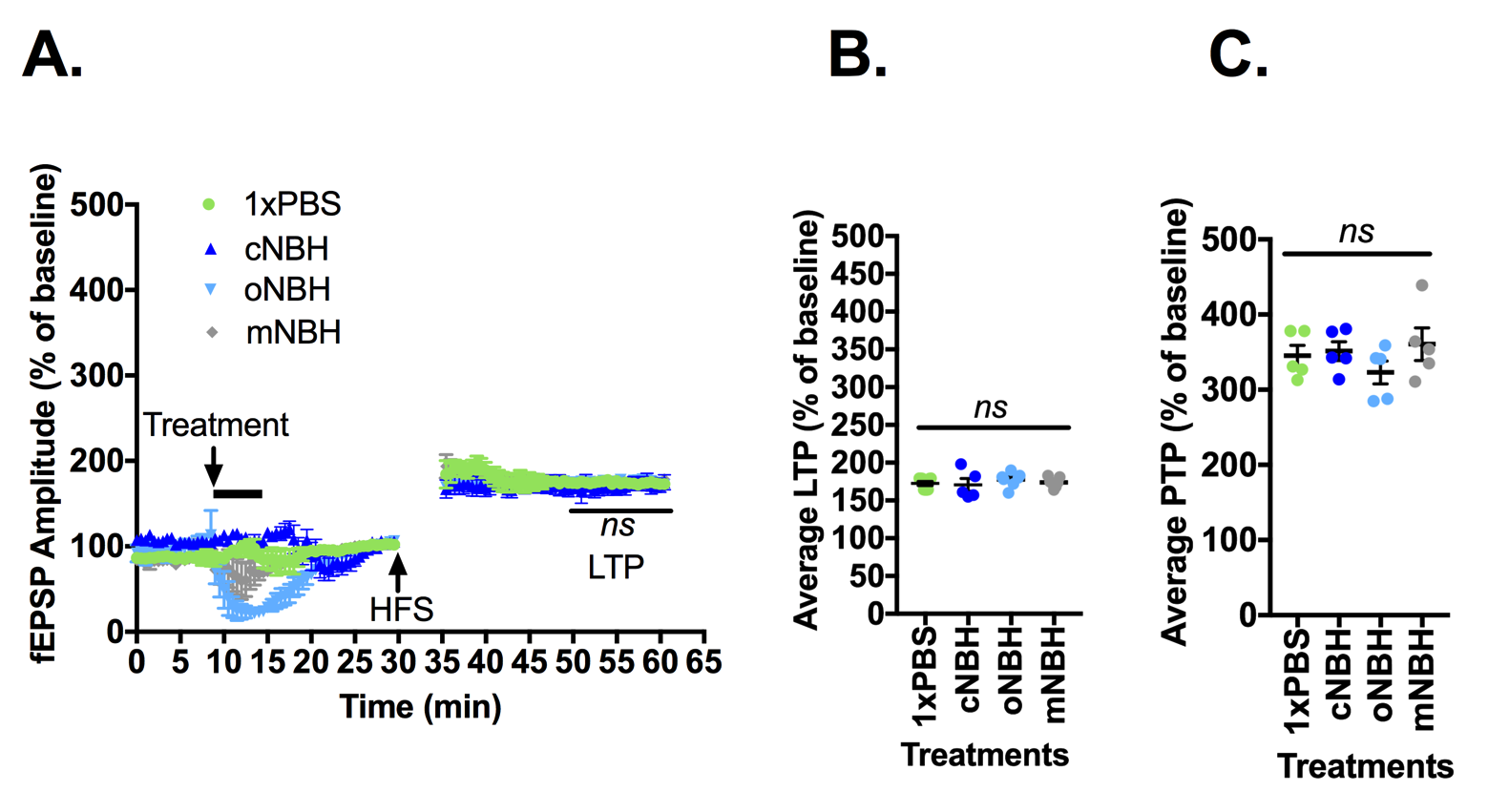

Supplement: S5 Fig — (A) LTP of WT mouse hippocampal slices following a five-minute treatment with 1x PBS negative technical control compared to ~0.5% (w/v in 1x PBS) cNBH after processing for size exclusion chromatography, as well as pooled oligomeric and monomeric fractions of NBH after size fractionation in 1x PBS. The five-minute treatment started after eight to 10 minutes of stable baseline. The high frequency stimulation (HFS) trains were applied following 30 minutes of baseline recordings. The average LTPs (B) and average PTPs (C) of slices treated with cNBH, oNBH, and mNBH were compared to those of slices treated with 1x PBS by One-way ANOVA with Dunnett’s correction for multiple comparisons. Results are presented as mean ± standard error of mean. ns = not statistically significant (p>0.05). (TIF) [file ppat.1007712.s005.tif]
